# Polymorphism mediated by electric fields: a first principles study on organic/inorganic interfaces†

**DOI:** 10.1039/d2na00851c

**Published:** 2023-03-23

**Authors:** Johannes J. Cartus, Andreas Jeindl, Anna Werkovits, Lukas Hörmann, Oliver T. Hofmann

**Affiliations:** a Institute of Solid State Physics, Graz University of Technology, NAWI Graz Petersgasse 16 8010 Graz Austria o.hofmann@tugraz.at

## Abstract

Organic/inorganic interfaces are known to exhibit rich polymorphism, where different polymorphs often possess significantly different properties. Which polymorph forms during an experiment depends strongly on environmental parameters such as deposition temperature and partial pressure of the molecule to be adsorbed. To prepare desired polymorphs these parameters are varied. However, many polymorphs are difficult to access within the experimentally available temperature–pressure ranges. In this contribution, we investigate how electric fields can be used as an additional lever to make certain structures more readily accessible. On the example of tetracyanoethylene (TCNE) on Cu(111), we analyze how electric fields change the energy landscape of interface systems. TCNE on Cu(111) can form either lying or standing polymorphs, which exhibit significantly different work functions. We combine first-principles calculations with a machine-learning based structure search algorithm and *ab initio* thermodynamics to demonstrate that electric fields can be exploited to shift the temperature of the phase transition between standing and lying polymorphs by up to 100 K.

## Abstract 2

This work investigates how external electric fields can manipulate interface polymorphism. We find it has strong impact and that it can be used to alter p-T phase diagrams.

## Graphical abstract

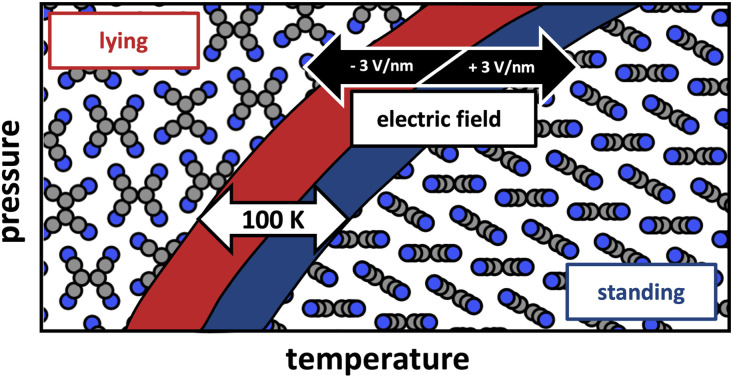

## 1 Introduction

The performance of nanotechnology applications often hinges on the properties of the interface between (metallic) contacts and the active organic material.^1,2^ Interestingly, not only the chemical composition of the material, but also its structure plays an important role for various properties, such as charge injection barriers^3–5^ or charge carrier mobilities,^6–9^*i.e.*, even if the constituents stay the same, the structure at the organic/inorganic contact has great impact.

Controlling the structure that forms at the interface is non-trivial, since most interfaces are usually grown out of thermodynamic equilibrium.^10–12^ But even when they are grown in thermodynamic equilibrium, the typically available handles – besides the choice of solvent^13,14^ - are deposition pressure and temperature, which can only be varied over a limited range. This range is on one side limited by the thermal stability of the adsorbate molecules and on the other side by the technical capability of the experimental setup. Another possible handle to affect the thermodynamic stability is to apply an electric field.^15–20^

Most experiments employing electric fields during growth have been performed for either (single) crystals^14,16,18,21–24^ or thin films.^19,25–30^ Therein, improved morphological characteristics of the prepared samples (increased grain sizes, crystal orientation, *etc.*) were found, which translated to improved bulk properties such as charge carrier mobilities,^16,26,27^ threshold voltages^16,31^ or thermoelectric properties.^32,33^ In these cases, the electric field either aligns the molecules in a specific direction or modifies the intermolecular interactions, which gives rise to changes in morphology or structure.^19,23^ However, for many modern nanotechnology applications, relevant properties are mostly determined by the interface, *i.e.*, the first layer of an (organic) molecule on a (metallic) substrate.^1,34–39^ Here, the application of an electric field would not only modify the intermolecular interactions, but also the interaction of the molecules with the substrate.

In this work, we use a combination of density functional theory and machine learning to study the example of an organic molecule on Cu(111) in an electric field. We demonstrate how the field affects the molecule–substrate and molecule–molecule interactions, which role charge transfer, screening and the (anisotropic) polarizability play, and how this affects the relative stability of different polymorphs. Here, we use the term polymorph to describe a specific packing arrangement of molecules on the surface.

This paper is organized as follows: first, we investigate how isolated TCNE molecules adsorb on the Cu(111) surface when electric fields are applied. Second, we explain how electric fields affect the molecule–substrate interactions for isolated molecules on the surface. Next, we extend this understanding to densely packed monolayers, thereby introducing molecule–molecule interactions. We demonstrate how their subtile interplay with molecule–substrate interactions is altered by the electric field. Finally, we discuss the implications of the altered interactions for the relative stability of polymorphs at different thermodynamic conditions.

## 2 Results and discussion

As model system for our investigations, we use the conjugated organic acceptor molecule tetracyanoethylene (TCNE) on a Cu(111) surface. This interface system is ideal for our study because its surface chemistry (in the absence of electric fields) is fairly well characterized, both experimentally^40–42^ and through simulations.^42–45^ Different polymorphs of TCNE on Cu(111) have been shown to exhibit substantially different properties such as the work function, which varies by up to 3 eV between polymorphs.^45^ TCNE is known to bond to the surface through a Blyholder-like interaction,^46^ comprising charge-donation from the metal into the molecular LUMO and back-donation from the molecular σ-system to the surface.^43^ Importantly, the resulting molecule–substrate interaction is strongly different for different adsorption geometries. Furthermore, like most conjugated organic molecules, TCNE is a molecule with different polarizabilities along different molecular axes.

We calculate the polarizabilities of TCNE along different molecular axes using density functional perturbation theory as implemented in FHI-aims^47,48^ (see Methods Section for details).

**Fig. 1:**
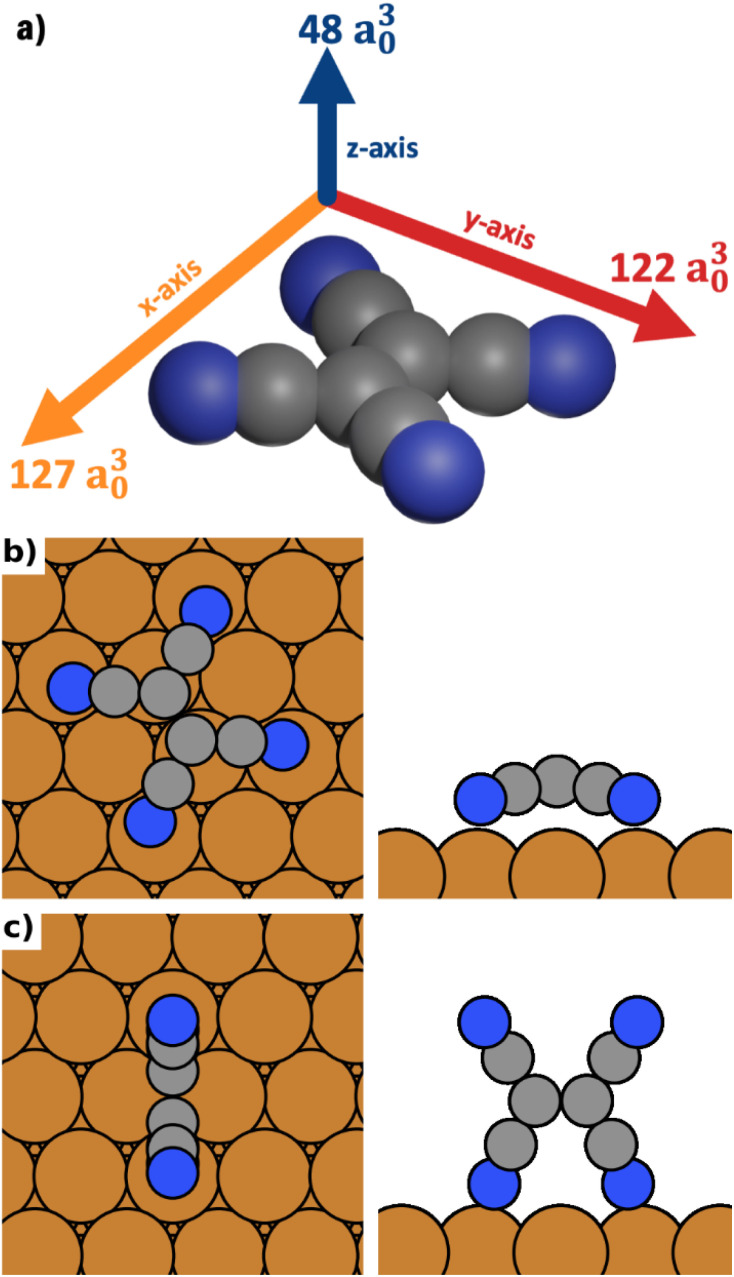
(a) TCNE and its polarizabilities in different directions (given by the length of the arrows and the number next to the arrows). (b) The most stable lying adsorption geometry (in absence of an electric field). (c) The most stable standing adsorption geometry (in absence of an electric field).

The results are visualized in Fig. 1a. We find that TCNE exhibits a large polarizability in the π-plane. We obtain a polarizability of 127 a^3^_0_ in the direction of the C=C bond (*x*-axis in Figure 1a) and 122 a^3^_0_ in the molecular axis perpendicular to the C=C bond (*y*-axis in Fig. 1a). The polarizability perpendicular to the π-plane (*z*-axis in Fig. 1a) is only about 40% of this value, 48 a^3^_0_. Since the polarizability is directly related to the change of the energy in an electric field, it follows from these values that TCNE molecules in gas phase will preferentially align with the π-plane parallel to the field.

### 2.1 Adsorption geometries in the electric field

Interface polymorphs are an assembly of molecules adsorbed on the surface. It is therefore instructive to study the adsorption geometry of the individual molecules. As TCNE bonds strongly to the Cu surface,^49^ it can be assumed that adsorption geometries in dense packed polymorphs remain similar to isolated-molecule geometries.

To understand how the electric field affects the adsorption geometries of isolated molecules, we start from the geometries obtained in earlier work by Egger *et al.* in absence of an external electric field.^43^ There, 11 distinct adsorption geometries were obtained by performing geometry optimizations from four different high-symmetry points (top, bridge, hcp-hollow and fcc-hollow) with the TCNE molecule placed in various orientations. Going beyond the method used by Egger *et al.*, we replaced the (then state-of-the-art) vdW^Surf^ dispersion correction^50^ with the more recent MBD-NL dispersion correction,^51^ which has been previously shown to yield adsorption energies in excellent agreement with experiment.^52,53^ Furthermore, we modelled the metal slab using 9 layers in order to obtain highly accurate adsorption energies. With these new settings, we re-optimized these geometries in a 6 × 6 super cell until the remaining forces fell below 0.01 eV Å^−1^. We find that our method has little impact on the geometries compared to the previously employed method and all geometries remain stable minima on the potential energy surface. Also, the relative adsorption energies are hardly affected. More details are given in ESI.†

We distinguish three categories of adsorption geometries based on which molecular axis is perpendicular to the surface (see Fig. 1a). There are four geometries with the *x*-axis perpendicular to the surface (“standing-*x*”), four with the *y*-axis perpendicular to the surface (“standing-*y*”) and three with the *z*-axis perpendicular to the surface (“lying”). Standing-*x* and standing-*y* category geometries differ in the orientation of the C=C backbone of the adsorbate relative to the surface. For *x*, it is perpendicular to the surface; for *y*, it is parallel. For the geometries of the lying category, all four nitrogen atoms are in contact with the surface. For the two standing categories only two nitrogen atoms bind to the surface, with the π-plane of the molecule being perpendicular to the surface.

We find that standing-*x* interacts with the surface so weakly, that it plays no role in the observable polymorphism. This agrees with earlier findings (see Egger *et al.*,^43^ where standing-*x* adsorption geometries were neglected in the structure search). We also find that standing-*x* and standing-*y* are affected by the field in a very similar way. Therefore, we only show differences between lying and standing-*y* hereafter. For the sake of brevity, we will also shorten the name of category “standing-*y*” to “standing”. We show the most stable adsorption geometries for the categories lying and standing in Fig. 1b and c.

To analyze how the adsorption geometries change in the presence of an electric field, we again relaxed all 11 previously obtained geometries in the presence of homogenous electric fields applied perpendicular to the Cu surface, using field strengths between −3 V nm^−1^ and +3 V nm^−1^. The sign of the field denotes the direction. Positive values correspond to a field whose field lines would point towards the interface for a positive test charge. We note that field strengths of this magnitude are larger than what is used in typical crystallization experiments,^19,23,54^ but they can, in principle, be experimentally realized, *e.g.*, in STM junctions.^55–57^ or in optical experiments.^58,59^ Such large field strengths are also considered in other theoretical works.^60–64^

After the relaxation, we observe that the adsorption geometries change only very little, with none of the atoms moving by more than 0.07 Å. Consequently, also the energy change associated with that geometry adaption is almost negligible, being less than 10 meV for all geometries.

In these relaxations, and throughout this work, the substrate is kept fixed. While relaxation of the substrate is expected to change the relative stability of adsorption geometries,^44^ this change (almost) constant and independent of electric field. Details are given in the ESI.† For the sake of simplicity, it is, therefore, neglected in the following discussion.

### 2.2 Molecule–substrate interactions: isolated molecules in the electric field

The relative stability of polymorphs is affected by intermolecular and molecule–substrate interactions. To study how the molecule–substrate interactions change in the field, we analyze the adsorption energy of isolated molecules. Because intermolecular interactions are not present for isolated molecules (by definition), the adsorption energy corresponds directly to molecule–substrate interactions.

We define the adsorption energy *E*_ads_ of an isolated molecule on the surface when an electric field of strength *F* is applied as

1 *E*_ads_(*F*) = *E*_sys_(*F*) − *E*_sub_(*F*) − *E*^vac^_mol_(*F*)

where *E*_sys_ is the energy of the combined molecule/metal system, *E*_sub_ is the energy of the Cu supercell, and *E*^vac^_mol_ is the energy of a TCNE molecule in vacuum. The energy of the system as well as of the components are evaluated in the presence of the electric field *F*. We note that negative values of *E*_ads_ correspond to exothermic reactions, *i.e.*, more negative values indicate stronger bonding to the surface.

While the calculation of the slab energy in the electric field is straightforward, for a TCNE molecule in gas phase the anisotropic polarizability must be considered. The energy of the molecule in the field varies with its orientation with respect to the field. At zero temperature it orients itself such that the molecular axis with the highest polarizability is aligned with the direction of the electric field. In case of TCNE, this is the *x*-axis in Fig. 1 (parallel to the C=C bond). This fact is important, as it means that the molecule must potentially reorient in the field to adsorb on the surface (*e.g.*, for the lying category). Additionally, the geometry of the molecule is deformed during adsorption, which (i) requires energy by itself, and (ii) gives rise to an adsorption geometry specific adsorbate dipole (hereafter called adsorbate dipole). This adsorbate dipole also changes in magnitude as the molecule is polarized by the electric field. We denote the component of the adsorption energy associated with all these geometric changes (reorientation, deformation, adsorbate dipole in the electric field) as *E*_geom_. It is obtained by calculating the energy difference between the deformed and the relaxed, optimally aligned adsorbate molecule in vacuum with the electric field applied. The remaining part of the adsorption energy, which stems from the bonding of the molecule to the substrate is denoted as *E*_bond_:

2 *E*_ads_(*F*) = *E*_geom_(*F*) + *E*_bond_(*F*).

In the following, we discuss how the interplay of *E*_geom_ and *E*_bond_ is changed by the field and how this, in turn, affects the relative stability of the adsorption geometries.

**Fig. 2:**
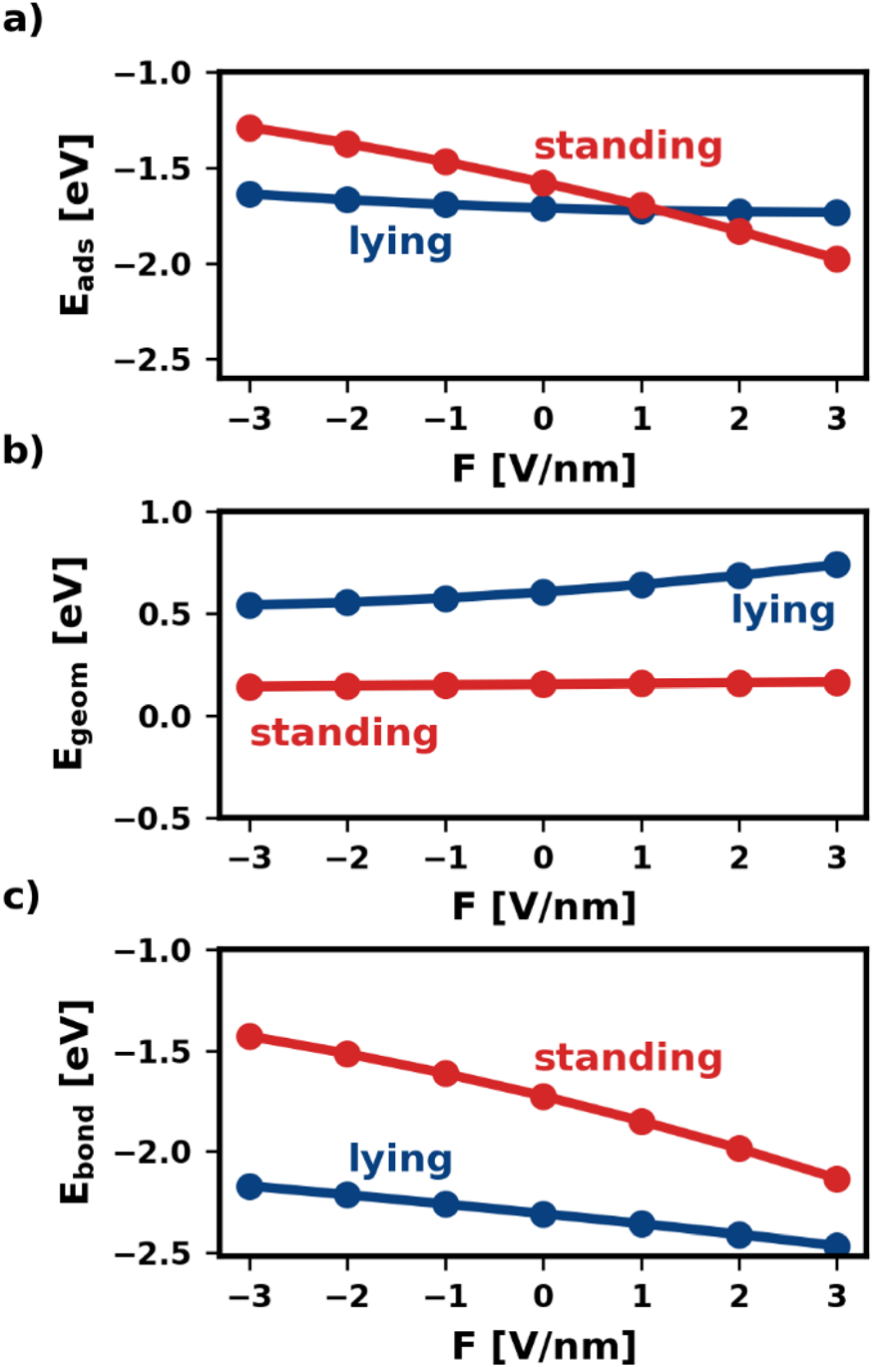
Adsorption energy of isolated molecules and their components as function of the electric field. Only the most stable adsorption geometry for every category is shown. (a) shows the total adsorption energy, (b) shows the component that corresponds to geometric changes during adsorption (reorientation, deformation, *etc.*), and (c) shows the component that stems from the bonding of the deformed and reoriented molecule with the substrate.

Fig. 2 shows the adsorption energy and its two components as a function of the electric field. We find that, within the same category, all geometries are similarly affected. Therefore, for the sake of simplicity, Fig. 2 shows only one representative geometry (most stable without electric field) for each of the categories lying and standing. For all other geometries, including the ones of the third category, standing-*x*, *E*_ads_ and its components are shown in the ESI.†

As shown in Fig. 2a, the electric field affects the adsorption energies of standing and lying categories very differently. We find that negative fields (*F* < 0) destabilize the adsorbed molecules, while positive fields stabilize the adsorption. However, the effect is quite strong for standing molecules, for which the adsorption energy changes by more than 1 eV between −3 V nm^−1^ and +3 V nm^−1^. Conversely, lying molecules are less affected, with *E*_ads_ differing only by 0.14 eV within the field strengths considered. As mentioned above, this is due to the interplay of *E*_geom_ and *E*_bond_. The two components respond differently to the electric field for different adsorption geometries. For the lying category, both components of the adsorption energy, *E*_geom_ and *E*_bond_, change by several 100 meV with the field. However, as these two contributions occur in similar magnitude, but with a different sign, these changes cancel each other for the adsorption energy. Conversely, for standing *E*_geom_ is almost constant, while *E*_bond_ changes even more than for lying.

The different changes in both *E*_bond_ and *E*_geom_ have multiple origins. As explained above, *E*_geom_ captures the reorientation and deformation of the molecule and the energy of the resulting adsorbate dipole in the field. The reorientation energy is rather small for standing adsorption geometries because the polarizability is very similar for both molecular axes that lie in the π-plane of the molecule (*x*-axis and *y*-axis). Standing molecules also deform very little during adsorption, resulting in a small deformation energy and adsorbate dipole. As a result, *E*_geom_ is small for standing adsorption geometries. For lying molecules, the situation is quite different. Lying molecules adsorb with the *z*-axis of the molecule perpendicular to the substrate surface (*i.e.*, in the direction of the field). The polarizability along the *z*-axis is much different from that along the *x*-axis, yielding a substantial reorientation energy. Furthermore, the molecule deforms significantly during adsorption. In the absence of an electric field *E*_geom_ corresponds entirely to the deformation energy. As can be seen in Fig. 2b at *F* = 0 V nm^−1^, the difference in deformation energy between the lying and the standing adsorption geometry amounts to approximately 0.5 eV. Thus, it accounts for most of the difference in *E*_geom_ between the lying and the standing geometry. The strong deformation lying molecules experience also gives rise to a large adsorbate dipole. Both the reorientation and the potential energy of the adsorbate dipole in the field give rise to strongly field-dependent contributions to *E*_geom_. As a result, *E*_geom_ is significantly impacted by the field for lying but hardly for standing.

The bonding energy *E*_bond_, on the other hand, is more strongly affected by the presence of the field (see Fig. 2c). While this is the case for both categories, the effect is particularly strong for the standing molecule. For the investigated fields, *E*_bond_ of the standing molecule changes by more than 1 eV. This is because the electric field not only affects how much charge is transferred between metal and molecule but also where (in the molecule) the charge density is localized.

To demonstrate this effect, we analyze how the electric field changes the electron density in the combined molecule/metal system. We define the charge density difference due to an electric field *F* as

3 Δ*ρ*(*F*) = *ρ*(*F*) − *ρ*(*F* = 0).

**Fig. 3:**
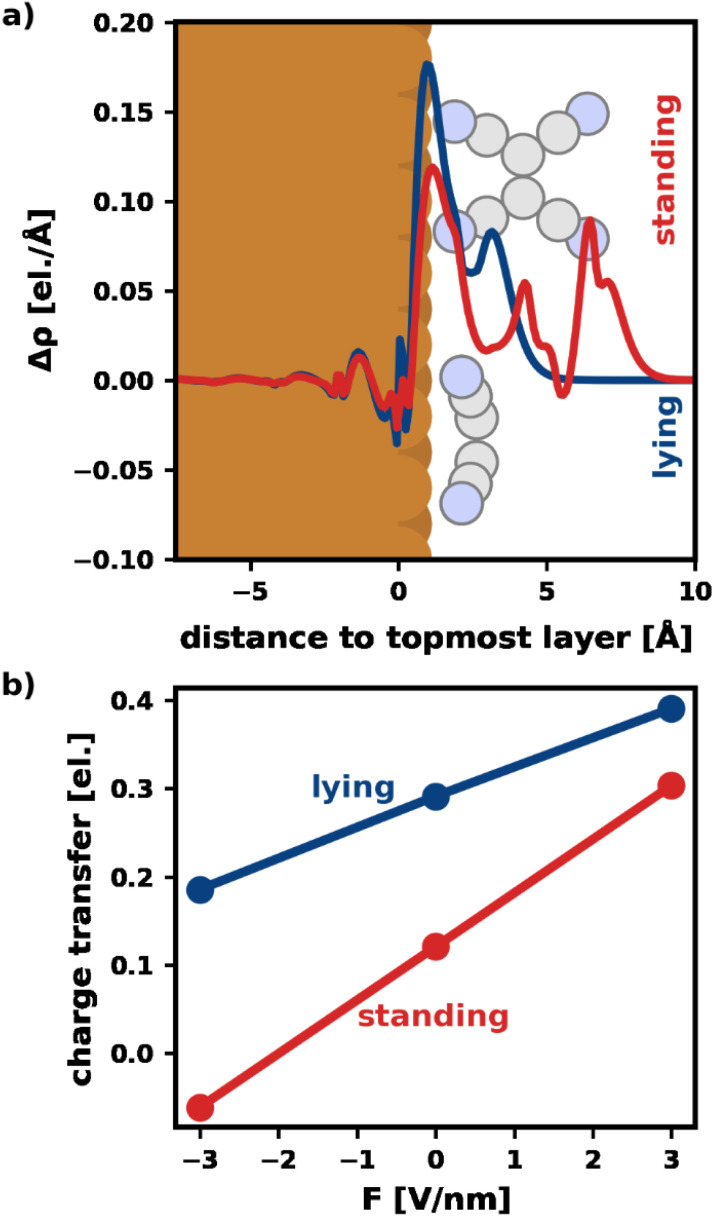
Visualization of the charge transfer. (a) Plane-averaged electron density difference between a field *F* = 3 V nm^−1^ and 0 V nm^−1^ for the most stable lying/standing adsorption geometries. The spatial extent of adsorption geometries of a lying and a standing molecule are indicated. (b) Net charge transfer into the molecule (calculated *via* Mulliken analysis) for the most stable lying/standing adsorption geometry.

Fig. 3a visualizes Δ*ρ* plane-averaged for an electric field of *F* = 3 V nm^−1^ for the most stable standing and lying adsorption geometry. (A corresponding plot for *F* = −3 V nm^−1^ is shown in the ESI†). As expected, there is an accumulation of charge on the surface of the substrate due to the polarization of the slab. Furthermore, the electrons in the standing molecule are more spread out, due to the spatial extent of the standing geometry in the direction of the field (*i.e.*, perpendicular to the surface). Standing molecules also exhibit a high polarizability in the direction of the field. As a result, it is energetically more favorable to shift electrons into the standing geometry, which can, therefore, benefit more from the field than the lying geometry.

To quantify the net charge transfer and its change with the electric field, Fig. 3b shows the net charge transferred from the substrate into the adsorbate (calculated *via* Mulliken analysis). In agreement with our findings for the charge density, we observe increased charge transfer for positive and decreased charge transfer for negative fields. The effect is stronger for the standing molecule, as can be seen from the slopes of the lines in Fig. 3b. In passing, we note that the change in net charge transfer to the adsorbate is primarily due to a change of donation from the substrate into the π-system of the adsorbates, whereas the back-donation of charge from the σ-system of the molecules into the substrate is far less affected by the field (ESI†).

In summary, we find that the electric field impacts the adsorption energy of isolated molecules in various ways: (i) the charges and dipoles of the interface system have a potential energy in the electric field that depends strongly on the adsorption geometry (*E*_geom_). (ii) To minimize this energy charges in the system are rearranged, which changes the bond of the adsorbates to the substrate. Both (i) and (ii) favor the standing adsorption geometry for field *F* > 0 V nm^−1^, thereby stabilizing it over the lying molecule.

### 2.3 Molecule–molecule interactions: tightly packed monolayers in the electric field

The structure of a tightly packed monolayer is the result of a delicate balance of molecule–substrate and molecule–molecule interactions.^65,66^ Having studied the molecule–substrate interactions through isolated molecules in the previous section, we now complete the picture by investigating the interplay of molecule–substrate and molecule–molecule interactions in tightly packed structures. It is important to note that both interactions are not independent of each other. Understanding how these interactions interact is a necessary requirement to effectively manipulate the interface polymorphism using electric fields.

The relative stability of polymorphs at zero temperature is determined by their adsorption energy per area. However, because it is more instructive for the following discussion, we use the adsorption energy per molecule hereafter. The adsorption energy per molecule of a polymorph in the electric field of magnitude *F*, whose unit cell contains *n* adsorbate molecules is defined as

4 *E*^ML^_ads_(*F*) = (*E*^ML^_sys_(*F*) − *nE*^vac^_mol_(*F*) − *E*_sub_(*F*))/*n*.

*E* ^ML^_ads_ is the difference between the energy of the combined monolayer/metal system *E*^ML^_sys_ and the energies of its components *E*_sub_ and *E*^vac^_mol_. *E*_sub_ is the energy of the Cu slab supercell and *E*^vac^_mol_ is the energy of a TCNE molecule in vacuum (both again with an electric field *F* applied).

To find the most stable standing/lying polymorphs, we performed a systematic structure search using SAMPLE.^67^ SAMPLE is a machine-learning-based structure search approach, which generates an exhaustive list of polymorphs (within a given coverage range) and predicts their energy. This is done using Bayesian linear regression, training on a small number of band structure calculations of polymorphs. Details regarding the training data and hyperparameters are given in the ESI.†

**Fig. 4:**
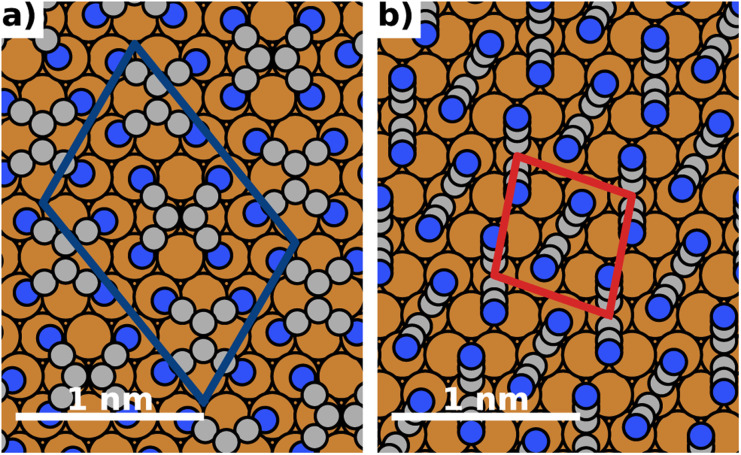
Most stable polymorphs (regarding energy per area) for *F* = 0 V nm^−1^ (no field). The coloured boxes show the unit cell. (a) Most stable lying polymorph, (b) most stable standing polymorph.

The energy differences between the polymorphs are very small and on the order of a few meV. Consequently, due to the differences in methodology, the best structure we find in this work (depicted in Fig. 4) is slightly different from the structure predicted in ref. 43. We emphasize, however, that they differ only in the relative orientation of molecules. Both the coverage and the adsorption energy are virtually indistinguishable from the structure predicted to be the best in ref. 43. A detailed comparison of the structures is given in the ESI.†

Analogous to the analysis in Section 2.2, we decompose the adsorption energy into three different components: *E*^ML^_geom_ is the geometric change during adsorption (reorientation and deformation, analogous to Section 2.2). *E*^ML^_formation_ is the interaction of the molecules and corresponds to the hypothetical process of the deformed molecules forming a monolayer in gas phase. *E*^ML^_bond_ which corresponds to the energy gained when the monolayer adsorbs on the surface:

5 *E*^ML^_ads_(*F*) = *E*^ML^_geom_(*F*) + *E*^ML^_formation_(*F*) + *E*^ML^_bond_(*F*)

*E* ^ML^_geom_ is again obtained as the energy difference between the deformed and the relaxed, optimally aligned adsorbate molecule. Similarly, *E*^ML^_formation_ is the difference between the deformed molecules in vacuum and the deformed molecules in the periodic unit cell of the free-standing adlayer (*i.e.*, without the substrate).

Furthermore, it is useful to decompose the total interface dipole *p*^ML^_int_ into the components arising from the molecules *p*^ML^_ads_ and from the bond *p*^ML^_bond_ to the surface:

6 *p*^ML^_int_(*F*) = *p*^ML^_ads_(*F*) + *p*^ML^_bond_(*F*).

**Fig. 5:**
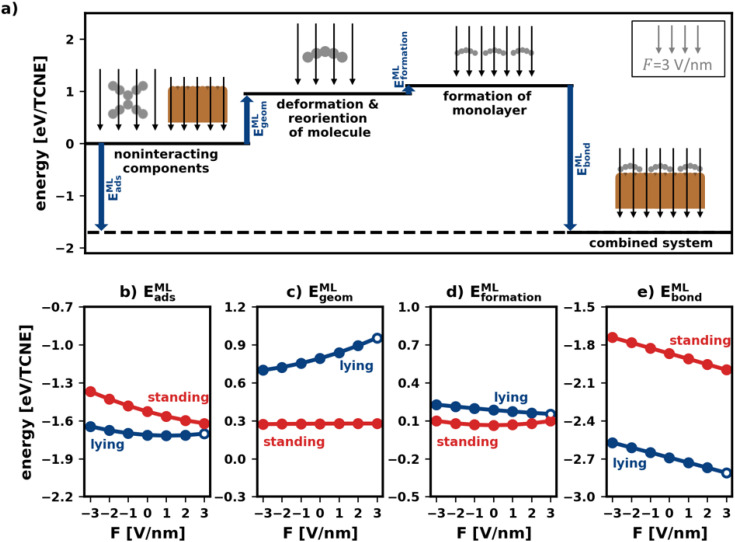
Decomposition of the adsorption energy of polymorphs. (a) Diagram of the case of the lying polymorph in an electric field of *F* = −3 V nm^−1^. The blue arrows show the adsorption energy and its components. (b–e) The adsorption energy and its components, all per molecule, as a function of the field for both the lying and the standing polymorph: (b) adsorption energy itself, (c) monolayer geometric change energy, (d) monolayer formation energy (e) monolayer bonding energy. The points in (b–e) that correspond to the energies shown as arrows in (a) are marked as a circle.

Fig. 5a visualizes the decomposition of the polymorph adsorption energy in a field of *F* = 3 V nm^−1^ for the lying polymorph schematically. Therein, the energy components are shown using arrows of corresponding magnitude. As can be seen, the bonding to the surface (*E*^ML^_bond_) is partially counteracted by both *E*^ML^_geom_ and *E*^ML^_formation_. This effect is dominated by *E*^ML^_geom_, because of the strong deformation of the lying molecules during adsorption (*cf.* Section 2.2).

To investigate how the field affects these energies, we depict them in Fig. 5b–e. The values are given in energy per molecule, rather than energy per area. This allows for easy comparison with the energies for the isolated molecule in Section 2.2/Fig. 2.

*E* ^ML^_geom_ (Fig. 5c) is an average of site-specific deformation and reorientation energies of the individual molecules. It is therefore not surprising that it behaves qualitatively like *E*_geom_ (*cf.*Fig. 2b): standing geometries are hardly affected while lying geometries are (de)stabilized by increasing negative (positive) fields.

Interestingly, the monolayer formation energy *E*^ML^_formation_ is very differently affected by the field for the two categories. However, as shown in Fig. 5d, its absolute changes with the field are so small that this is, in practice, almost inconsequential. A more detailed discussion is given in the ESI.†

*E* ^ML^_bond_ is the largest component of *E*^ML^_ads_ (see Fig. 5e). Seemingly, in the absence of an electric field, the molecules bond more strongly to the surface when densely packed than when they are alone. We emphasize, however, that the absolute values of *E*^ML^_bond_ in Fig. 5e should not be compared with the values from Fig. 2c because the adsorption geometries in the tightly packed layer are different from those considered above. Rather, the important finding here is that when comparing isolated and densely packed molecules, the change of *E*^ML^_bond_ with field changes qualitatively for upright standing molecules: the field dependence is now smaller than it was before. This is not quite unexpected, since packing molecules more densely generally increases the dielectric constant of the layer and reduces its polarizability.^3^ Interestingly, in the tightly packed layers the polarizability of both the upright standing and the flat-lying structures (given by the derivative of the energy with respect to the field) are approximately equal.

Taken together (Fig. 5b), we conclude that the impact of the field for tightly packed molecules is quite different from the impact the field has on isolated moieties. Here, this has a profound impact: while one could have expected from the initial treatment of isolated molecules, that upright-standing polymorphs become significantly stabilized by positive fields, the “collective” effects from tightly packed molecules significantly mitigate the prospective gain.

### 2.4 Temperature & pressure dependence: electric fields as handles in experiment

To achieve the best control over the interface polymorphism in an experiment, having multiple, independent handles is desirable. Therefore, we investigate how the commonly used handles deposition temperature, deposition pressure, and electric field interact and influence the interface polymorphism. We do this using *ab initio* thermodynamics,^68–70^ which has been applied to many different interface systems to analyze the temperature and pressure dependencies. Because most studies are done without electric fields present, the field-dependent terms, to the best of our knowledge, cannot be found in literature. Because it is *a priori* unclear how large they are, it is necessary to briefly revisit the *ab initio* thermodynamics treatment and the commonly made approximations. In the following, we show that for TCNE/Cu(111), the field-dependent terms are generally small, but highlight that this is not necessarily always the case.

*Ab initio* thermodynamics, as described *e.g.*, by Reuter *et al.*,^68,71^ starts with the assumption that interface structures are grown in thermal equilibrium. The adsorbed molecules are in thermodynamic equilibrium with an infinite reservoir of adsorbate molecules in gas phase. Together, the reservoir and the interface make up a thermodynamic system with a fixed number of particles. The surface and the reservoir share the same temperature, as they are in thermodynamic equilibrium. The pressure we consider in this thermodynamic system is the partial pressure of the adsorbate molecules being evaporated into the chamber for deposition. Furthermore, here we assume that the electric field *F* is constant and homogeneous within the whole gas phase reservoir.

To find which polymorph forms for given conditions one must compare the thermodynamic stability of possible polymorphs. The thermodynamic potential that determines the relative stability of polymorphs in this regime is the Gibbs free energy of adsorption per area:7
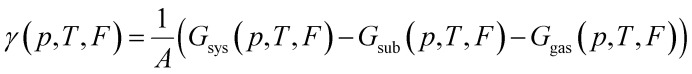
where *G*_sys_, *G*_sub_ and *G*_gas_ are the Gibbs free energies of the combined monolayer/metal system, the substrate, and the adsorbate molecules in gas phase. The area of the unit cell of the polymorph is denoted as *A*.

It is common in literature,^68,70–73^ to neglect the contribution of the configurational entropy and mechanical work to *γ*. Both terms are unaffected by an eventual electric field. It is also customary to neglect contributions from vibrations. This is, in principle, a valid approximation when the interaction between adsorbate and substrate does not affect the vibrations significantly. Indeed, it has been shown that the vibrational entropy of the pristine substrate and the substrate in the combined monolayer/metal system tends to cancel.^68,70–72,74^

Conversely, the vibrations of the molecules on the substrate can differ substantially from the molecules in the gas phase, especially when charge-transfer at the interface occurs.^73^ For the special case of TCNE/Cu(111), it was shown in previous work^44,45^ the zero-point energy of the adsorption geometries differs only very little when there is no field applied.

In principle, the field changes the charge state on the surface considerably (see Fig. 3), and since different adsorption geometries are differently affected by the field, one could expect that the (relative) vibrational energy changes with the field. While this is certainly the case in general, for our example of TCNE on Cu(111) we show in the ESI† that the charge state (coincidentally) does not play a noticeable role. Furthermore, we also find that the change of the zero-point energy of TCNE molecules with the electric field is small regardless of molecule orientation relative to the field, as we show in the ESI.† Thus, for the present work, we, therefore, neglect the contributions from vibrations of molecules on the surface as well, *i.e.*, *G*_sys_(*p*,*T*,*F*) ≈ *E*^ML^_sys_(*F*).

*G*_gas_ is often approximated using the ideal gas model.^69,70^ In the presence of an electric field, however, some components become field-dependent. For a polymorph containing *n* TCNE molecules in its unit cell the Gibbs free energy can be expressed through the chemical potential of the gas phase reservoir:

8 *G*_gas_(*p*,*T*,*F*) = *n*·*μ*_gas_(*p*,*T*,*F*).

To calculate the chemical potential of the gas phase, the translational, roto-vibrational, and electronic degrees of freedom of TCNE must be considered:

9 *μ*_gas_(*p*,*T*,*F*) ≈ *μ*^elec^_gas_(*p*,*T*,*F*) + *μ*^trans^_gas_(*p*,*T*,*F*) + *μ*^rot^_gas_(*p*,*T*,*F*) + *μ*^vib^_gas_(*p*,*T*,*F*),

In eqn (9), it was assumed that electronic, rotational, and vibrational motion may be treated separately, given that they occur on different time scales. The translation is not affected by the field and the ideal gas model can be applied: *μ*^trans^_gas_(*p*,*T*,*F*) = *μ*^trans^_ideal_(*p*,*T*). For the electronic energy, density functional theory already provides a high-quality description: *μ*^elec^_gas_(*T*,*F*) ≈ *E*^vac^_mol_(*T*,*F*). As discussed above, the energy depends on the orientation of the molecule due to the anisotropic polarizability. In an ensemble average, the orientations of the TCNE molecules follow a Boltzmann distribution, which results in a temperature dependence of the electronic energy. For the special case of TCNE, this temperature dependence is small enough to be neglected. (*i.e.*, *μ*^elec^_gas_(*p*,*T*,*F*) ≈ *E*^vac^_mol_(*F*)). We note, however, that for molecules with even more anisotropic polarizabilities, this may not be the case.

A second effect of the anisotropic polarizability is that it creates a potential barrier for the rotation, *i.e.*, the rotation of the molecule becomes hindered. For TCNE, once again we find that this hindrance is negligible at relevant temperatures, as shown in the ESI.† Therefore, we continue to use the ideal gas model for the rotational degrees of freedom: *μ*^rot^_gas_(*p*,*T*,*F*) ≈ *μ*^rot^_ideal_(*p*,*T*). However, also here, for molecules with larger, more anisotropic polarizabilities, this term can become significant.

Finally, vibrations of the molecule give rise to a dipole, which has a potential energy in the field (analogous to the adsorbate dipole, but much smaller). As mentioned above, we find that the change in the zero-point energy of the vibrations is small regardless of the orientation of the TCNE molecule relative to the field. Therefore, for TCNE it is appropriate to continue using the ideal gas model for vibrational degrees of freedom, *i.e.*, *μ*^vib^_gas_(*p*,*T*,*F*) ≈ *μ*^vib^_ideal_(*p*,*T*).

Taken together, we model the chemical potential of the TCNE gas phase in the electric field *F* as

10 *μ*_gas_(*p*,*T*,*F*) ≈ *E*^vac^_mol_(*F*) + *μ*^trans^_ideal_(*p*,*T*) + *μ*^rot^_ideal_(*p*,*T*) + *μ*^vib^_ideal_(*p*,*T*),

Based on these approximations, the only effect of the electric field that enters the *ab initio* thermodynamic treatment is the change of the electric energy due to polarization and the alignment of the molecules in the field.

### 2.5 Application: shifting the phase boundary between lying and standing polymorphs

In this section, we apply our findings from Section 2.4 to study the combined effects of different experimental conditions on the polymorphism. It is instructive to consider the case without an electric field (*F* = 0 V nm^−1^) first, *i.e.*, to only vary pressure and temperature. In earlier work, Egger *et al.*^43^ provided a pressure–temperature phase diagram in their supporting information that shows the most stable category (lying/standing) for different pressures and temperatures. To reproduce this phase diagram and extend it to different electric fields, we predicted the energies for different fields using the SAMPLE approach.^67^ This is done by training energy models with band structure calculations of 200–300 different polymorphs (all details in the ESI†). Separate energy models were trained for all external electric fields between −3 V nm^−1^ and +3 V nm^−1^.

**Fig. 6:**
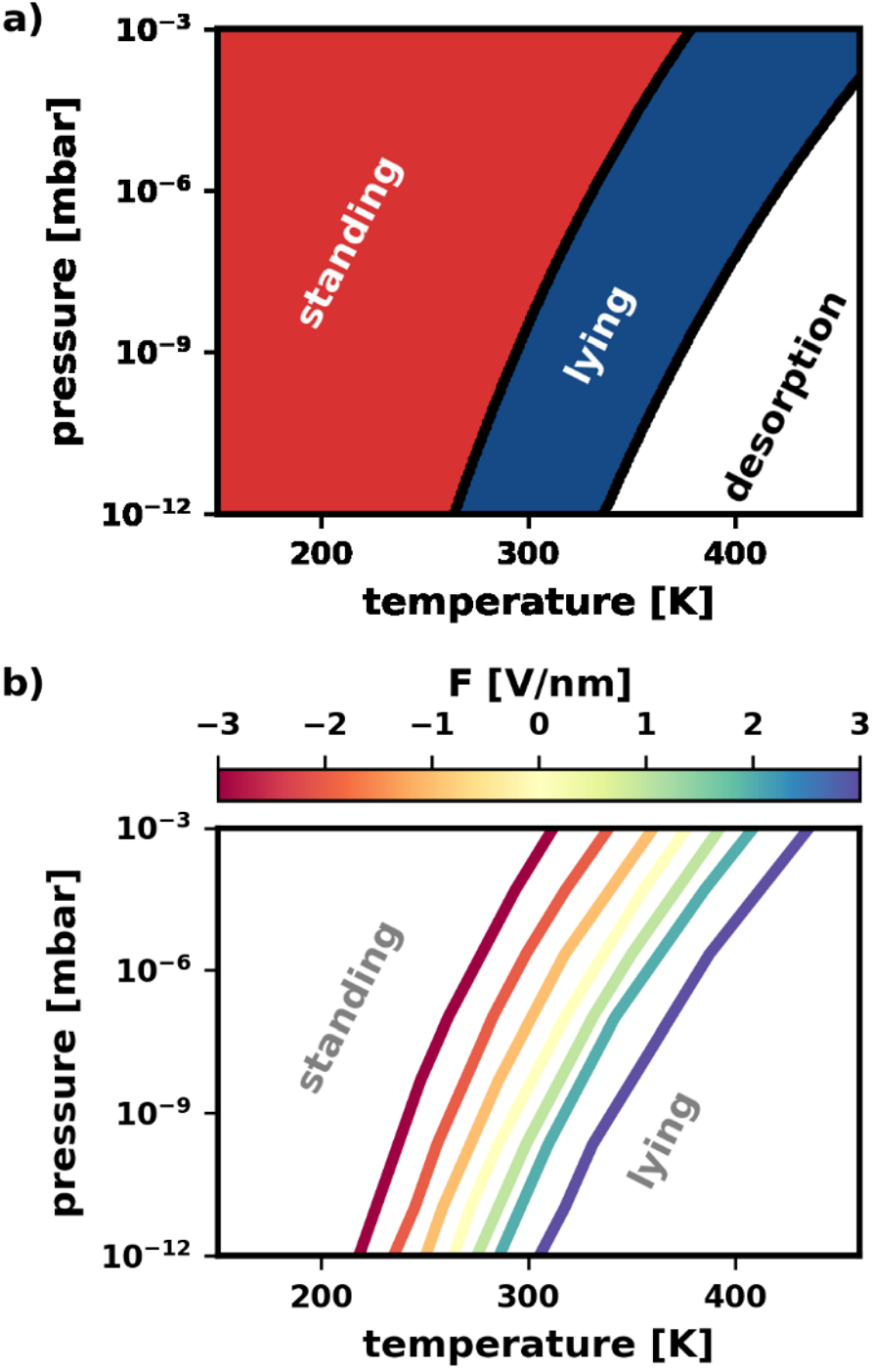
Phase diagram showing the relationship between most stable polymorph category and pressure, temperature, and electric field. Pressure refers to the partial pressure of the TCNE in gas phase. (a) Pressure–temperature phase diagram for *F* = 0 V nm^−1^ (*i.e.*, no field applied). The red phase corresponds to standing polymorphs, the blue phase to lying polymorphs. (b) Change of the pressure–temperature phase boundary between standing and lying for different electric fields.

As can be seen in Fig. 6a, for low temperatures and high pressures more tightly packed structures form. This favors the standing polymorphs. With increasing temperature, packing density becomes less important relative to bonding to the surface, which yields a transition from standing to lying polymorphs. Finally, for even higher temperatures and lower pressures, the molecules desorb. In passing, we note that the qualitative agreement with the phase diagram from Egger *et al.*^43^ is excellent, despite small quantitative differences due to the different methodologies.

Finally, we demonstrate that the electric field can be used to change the way pressure and temperature affect the polymorphism. Of particular interest is the phase boundary between standing and lying structures. Knowing that the electric field can tune the relative stability of polymorphs, we expect the electric field to have a significant impact.

Fig. 6b confirms this expectation. Therein, the location of the phase boundary for different electric fields is explicitly plotted. When fields *F* > 0 V nm^−1^ are applied, the boundary is shifted to higher temperatures. Reversing the field to *F* < 0 V nm^−1^, we observe the boundary to move towards lower temperatures. The shape of the boundary remains qualitatively the same for all fields, with a slight increase in curvature for more intense fields *F* > 0 V nm^−1^. In other words, a wider range of temperature/pressure combinations can be used to “trigger” the phase transition when applying these fields. Finally, it is worth noting, that the shift of phase boundary is quite substantial: according to our calculations the transition temperature can be varied by about 100 K for the pressure range of 10^−12^ to 10^−3^ mbar. As the predictions are somewhat dependent on the errors made by the prediction of the adsorption energies *via* SAMPLE, we provide an analysis of the error propagation in the ESI.†

The above demonstrates that the electric field is a quite useful handle to manipulate the pressure–temperature phase diagram. At the same deposition pressure, it enables the preparation of *e.g.*, a lying polymorph at much lower temperatures. In other words: the electric field stabilizes the lying structures in a thermodynamic region where they would usually not be accessible.

## 3 Conclusions

We investigated the effect of external electric fields on the relative stability of polymorphs of organic/inorganic interfaces using the example of tetracyanoethylene (TCNE) on Cu(111). TCNE is chemisorbed on the substrate and exhibits two distinctly different categories of polymorphs: “lying” and “standing”. We demonstrate that the geometric and chemical differences between the two categories can be exploited to manipulate the thermodynamic properties of this system in using external electric fields.

For the investigated field intensities, we find that the adsorption geometries of individual molecules change only very little. However, the adsorption geometries of the adsorbing molecules affect the relative stability of the polymorphs significantly through the energy of the adsorbate dipole in the field. Some adsorption geometries allow for a more beneficial distribution of charges in the field than others.

Furthermore, we found that electric fields can tune the molecule–substrate interactions through manipulation of the charge transfer into the molecules. This changes the bond dipole of the molecules, increasing the bond with the surface but also the intermolecular repulsion due to dipole–dipole interactions.

The combination of both effects enables manipulating the relative stability of lying and standing polymorphs substantially: in detail, we predict that the phase boundary between lying and standing polymorphs can be shifted in a temperature range of about 100 K when applying electric fields between −3 V nm^−1^ and 3 V nm^−1^ in a pressure range that is experimentally relevant (10^−12^ to 10^−3^ mbar). A permanent dipole is not required to use external electric fields to impact the polymorphism if the dipoles created during adsorption are sufficiently distinct. However, how strong the effect of an electric field is, of course, depends on the system. This is because effects other than the anisotropy of the polarizability, *e.g.*, the nature of the bond to a (possibly partially passive) substrate, can play an additional role. Regardless, introducing electric fields as an additional lever in deposition experiments opens doors to conduct growth in technically less challenging temperature and pressure regions or even to stabilize polymorphs at hitherto inaccessible regions.

## 4 Computational methods

All band-structure calculations were done with FHI-aims^47^ using the PBE^75^ exchange-correlation functional with the MBD-NL dispersion correction^51^ scheme. FHI-aims relies on numerically tabulated, atom-centered orbitals and supports both periodic and open boundary conditions. Except for the free molecule in vacuum, which was modeled with open boundary conditions, all calculations were done using periodic boundary conditions employing the repeated-slab approach. The metal slab was modelled with 9 metal layers, with a vacuum region of more than 55 Å above it. The electric field is modelled by applying a sawtooth potential with a potential jump in the vacuum region.^76^ The polarizability tensor for the system is calculated using *ab initio* density functional perturbation theory.^48^

Our SCF convergence settings require the energy to change by less than 10^−6^ eV and the electron density by less than 10^−2^ el. between subsequent SCF iterations. We used a Gaussian occupation scheme with a broadening of 0.1 eV for the states. The reciprocal space was sampled by a generalized Monkhorst–Pack grid^77,78^ with a maximal spacing of Δ*k* = 2π/80 Å^−1^.

The numerical settings are based on prior work with Cu(111)/TCNE by some of us.^43^ In detail, we used tight species defaults, as supplied by FHI-aims (prior to 2020) for the carbon and the nitrogen atoms. For the first three layers of the Cu substrate, we used settings that were slightly modified from the shipped “tight” defaults. Specifically, the onset of the cutoff potential is increased to 4.6 Å, the radial multiplier is reduced to 1, the 5g-basis function is omitted and the *basis_dep_cutoff* is reduced to 10^−3^ Å. The settings for the remaining substrate layers are based on FHI-aims’ ‘light’ species defaults for Cu, with the following changes: the *basis_dep_cutoff* is reduced to 10^−3^ Å, the angular grid divisions are changed to division 0.3478 50; division 0.6638 110; division 0.9718 194, and only the minimal basis with additional 4p functions are used. All settings are carefully tested, see ESI.†

## Data availability

The band structure calculations of this study are openly available in the NOMAD repository (https://doi.org/10.17172/NOMAD/2023.03.16-1).

## Author contributions

Johannes J. Cartus: conceptualization, methodology, software, validation, formal analysis, investigation, data curation, writing – original draft, writing – review & editing, visualization. Andreas Jeindl: methodology, investigation, formal analysis, writing – review & editing, data curation. Anna Werkovits: methodology, investigation, formal analysis, writing – review & editing, visualization. Lukas Hörmann: methodology, investigation, formal analysis, writing – review & editing, visualization. Oliver T. Hofmann: conceptualization, resources, investigation, project administration, funding acquisition, supervision, writing – review & editing.

## Conflicts of interest

The authors have no conflicts of interest to declare.

## Supplementary Material

NA-005-D2NA00851C-s001
