# Supplementary material for: Polymorphism mediated by electric fields: a first principles study on organic/inorganic interfaces
Source: Nanoscale Adv. 2023 Mar 23;5(8):2288–98. doi: 10.1039/d2na00851c (PMC10089127; doi:10.1039/d2na00851c)
Supplement: NA-005-D2NA00851C-s001 [file NA-005-D2NA00851C-s001.pdf]

Electronic Supplementary Material (ESI) for Nanoscale Advances.
This journal is © The Royal Society of Chemistry 2023

Supporting information to

Polymorphism mediated by electric fields: A first principles study on organic/inorganic interfaces

Johannes J. Cartus,[1] Andreas Jeindl,[1] Anna Werkovits,[1] Lukas Hörmann,[1] and Oliver T. Hofmann[1*]

[1] *Institut für Festkörperphysik, Technische Universität Graz, Petersgasse 16, 8010 Graz, Austria*

* *Corresponding author. E-mail: cartus@tugraz.at*

S1 Convergence tests for the adsorption energy

S1.1 Custom basis set

We used the same custom basis set as Egger et al.[1] It is based on the species defaults supplied with FHI-aims (prior to 2020). In detail, we used tight species defaults, as supplied by FHI-AIMS (prior to 2020) for the carbon and the nitrogen atoms. For the first three layers of the Cu substrate we used settings that were slightly modified from the shipped "tight" defaults. Specifically, the onset of the cutoff potential is increased to 4.6 Å, the radial multiplier is reduced to 1, the 5g-basis function is omitted and the and the basis_dep_cutoff is reduced to 1×10^{-3} Å. The settings for the remaining substrate layers are based on FHI-aims' "light" species defaults for Cu, with the following changes: The basis_dep_cutoff is reduced to 1×10^{-3} Å, the angular grid divisions are changed to division 0.3478 50; division 0.6638 110; division 0.9718 194, and only the minimal basis with additional 4p functions are used. Using a 6×6 super cell and a Cu slab of 5 layers, we compared the adsorption energies of the best adsorption geometries found by Egger et al. for each of the categories lying and standing, as we define them in the main paper. In detail, we compare a slab with the basis for all atoms set to "tight" as supplied by FHI-aims (prior to 2020) to a slab with the basis settings as suggested by Egger et al. (described in the Method Section of the main paper). We find that the adsorption energies differ by no more than 6.5 meV between the two basis sets and the difference in adsorption energy between the standing and lying differs by no more than 4.2 meV between the two basis sets.

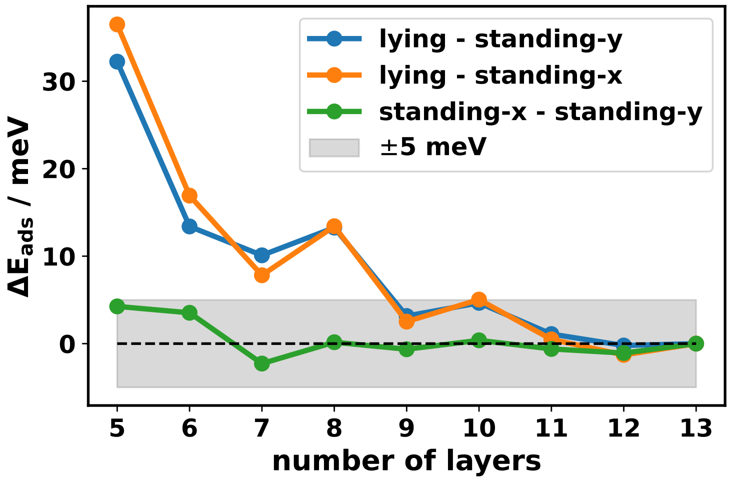

Figure S1: Layer convergence of the difference in adsorption energy for molecules in different adsorption geometries

S1.2 Layer convergence

We converged the difference in adsorption energies for isolated molecules in a 6×6 super cell (see Figure S1). The molecular geometries were the best adsorption geometries found by Egger et al[1] for the categories described in the main paper (standing-x, standing-y and lying). We employed the mixed basis set described in the previous section. Therein, we used settings based on the "tight" defaults for the three topmost layers and the settings based on the "light" defaults for all additional layers. We found that the difference falls to 5.1 meV for 9 layers of Cu.

S2 Adsorption geometries

S2.1 Adsorption geometries found by Egger et al.

Figure S2 shows the adsorption geometries of TCNE on Cu(11) we employed in this work. They were originally obtained by Egger et al.[1] who performed geometry optimizations of isolated molecules in a 6×6 Cu supercell. As starting points, they placed the molecules at different high symmetry points of the substrate in different molecular orientations. The geometries shown below, were re-optimized as described in the following section.

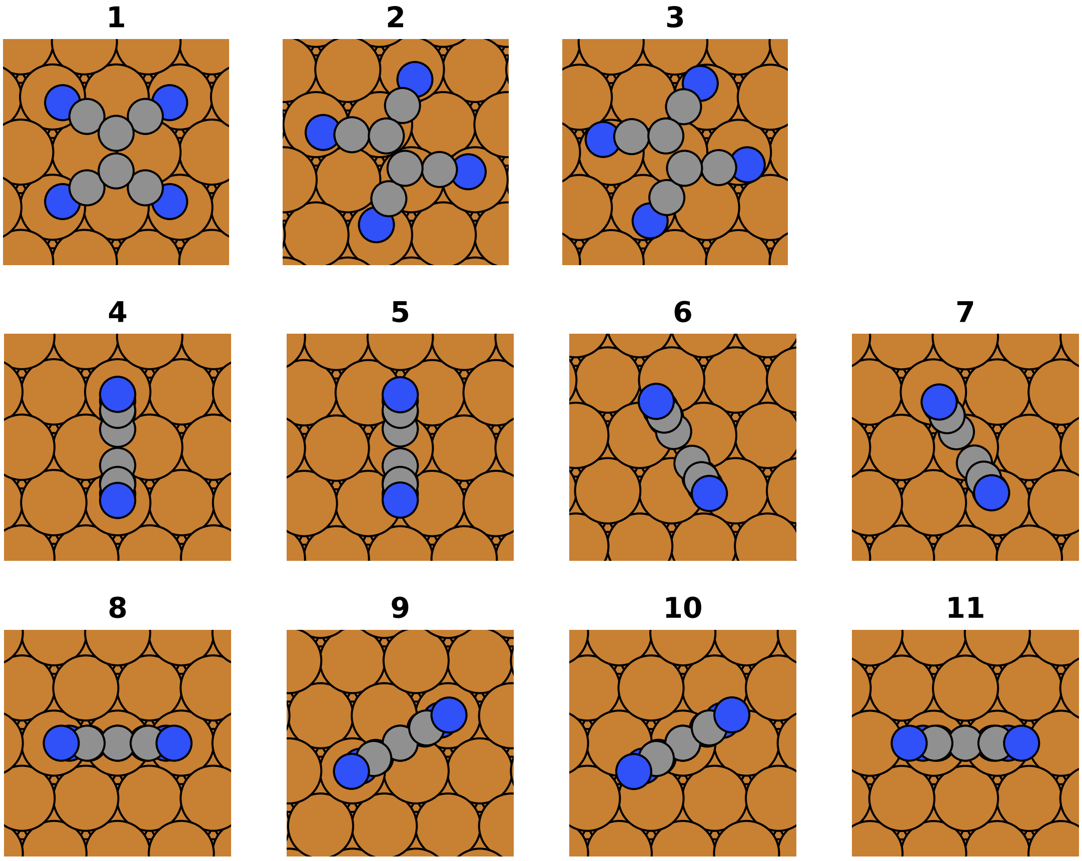

Figure S2: Adsorption geometries of TCNE on Cu(11) we employed in this study. They are categorized as follows: 1-3 are lying, 4-8 are standing-y and 9-11 are standing-x.

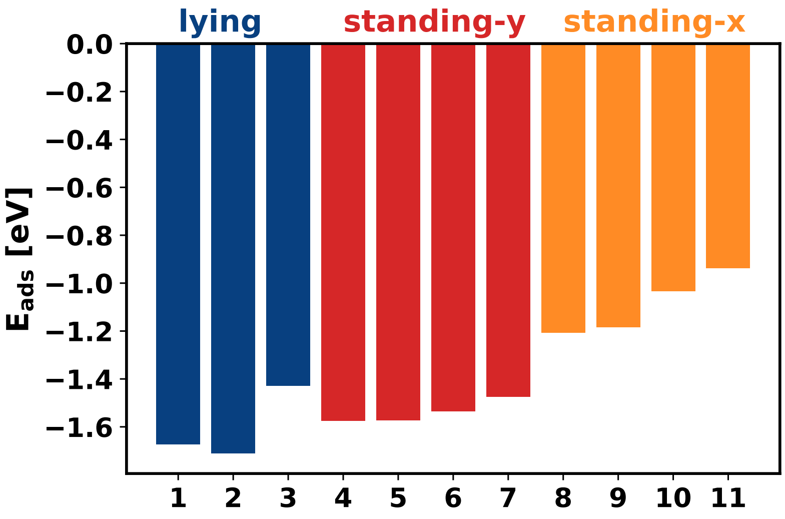

Figure S3: Adsorption energies for all adsorption geometries obtained by Egger et al., re-optimized with the methodology employed in this paper.

S2.2 Adsorption energies and re-relaxation with our methodology

Applying our methodology (different numbers of layers and different dispersion correction) we relaxed the adsorption geometries obtained by Egger et al. until the forces fell below 1×10^{-2} eV Å^{-1}. The adsorption energies change very little during the relaxation, with atoms moving by less than 0.04 Å and the adsorption energies changing by less than 3 meV (with respect to the starting point of the relaxation, also calculated with the methodology employed in this paper).

With the methodology we employed, the absolute values of the adsorption energies change by up to 200 meV compared to the values obtained by Egger et al. Changes of this magnitude are not uncommon for different methods. For comparison: Hörmann et al.[2, 3] benchmarked the performance of several functionals for PTCDA on Ag(111) and obtained a difference of ca. 840 meV between PBE+vdW^{Surf} and PBE+MBD-NL for the adsorption energy of the most stable adsorption geometry. Notably, the relative stability of the minima is much less dependent on the choice of computational method. As a result, we find the ranking of the adsorption geometries with respect to their adsorption energies changes slightly, but the ranking of the categories remains unchanged. The adsorption energies for the individual adsorption geometries are shown in Figure S3. The most stable adsorption geometry for lying is adsorption geometry 2 and the most stable for standing is adsorption geometry 4. These are the geometries that are featured in the figures in the main paper.

S2.3 How do the adsorption energies change with the field?

In Figure S4 we show the adsorption energy of the geometries of the isolated molecules as a function of the field. As discussed in the main paper, we find that the adsorption energy of the geometries behaves qualitatively similar to energies of other geometries in the same respective categories.

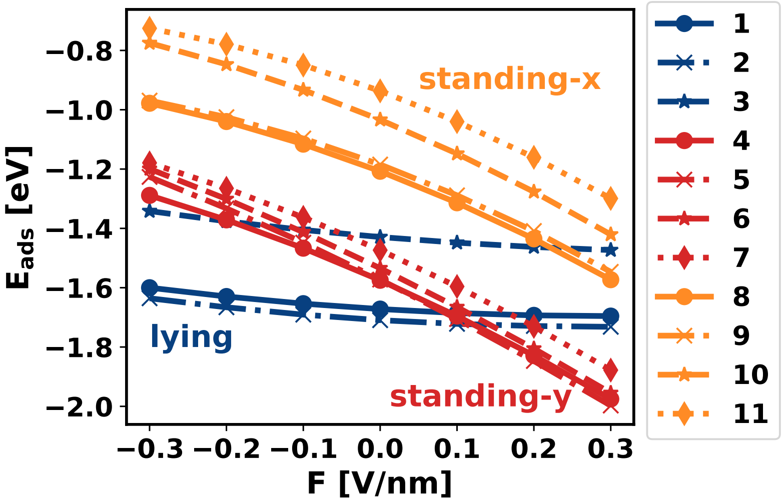

Figure S4: Adsorption energy of the isolated molecule in various adsorption geometries as a function of the electric field F.

S2.4 How do the adsorption energies change with the field?

The deformation of the substrate has been observed to have an impact on the relative stability of lying and standing adsorption geometries of TCNE on Cu(11).[4] However, as is shown in Figure S5, the adsorption energy difference between adsorption geometries where the substrate was kept fixed and those where it had been relaxed is almost constant for different electric fields. For the results in Figure S5 the most stable lying and standing geometries were re-optimized with different electric fields applied while also allowing the first two substrate layers to relax unit the forces fell below $1 \times 10^{-2}\,\mathrm{V\,Å^{-1}}$.

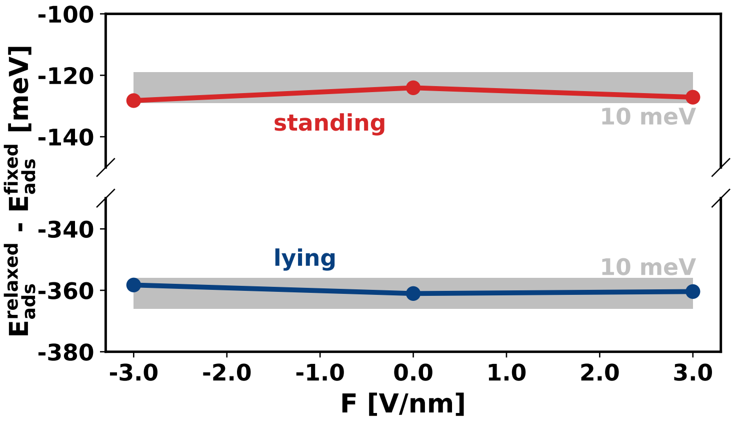

Figure S5: Impact of substrate relaxation. The adsorption energy difference between adsorption geometries with a relaxed substrate and a fixed substrate as a function of the electric field F for the most stable lying and standing adsorption geometry.

S3 Charge transfer: Donation and back donation

To analyze the charge transfer mechanisms for the isolated orbitals on the surface, we projected the density of states onto corresponding molecular orbitals (MODOS).[5] From this we

calculated the change in orbital occupation and subsequently the amount of donation (charge transferred into the molecule) and back donation (charge transferred into the substrate). The result is visualized for various field strengths in FigureS6. For standing, we find that the back donation is affected very little by the field, whereas the (forward) donation changes substantially with the field. As opposed to this, the forward and backward donation both change in similar amounts. As a result, the change in net charge transfer with the field (i.e., the slope of the curve) is smaller for lying.

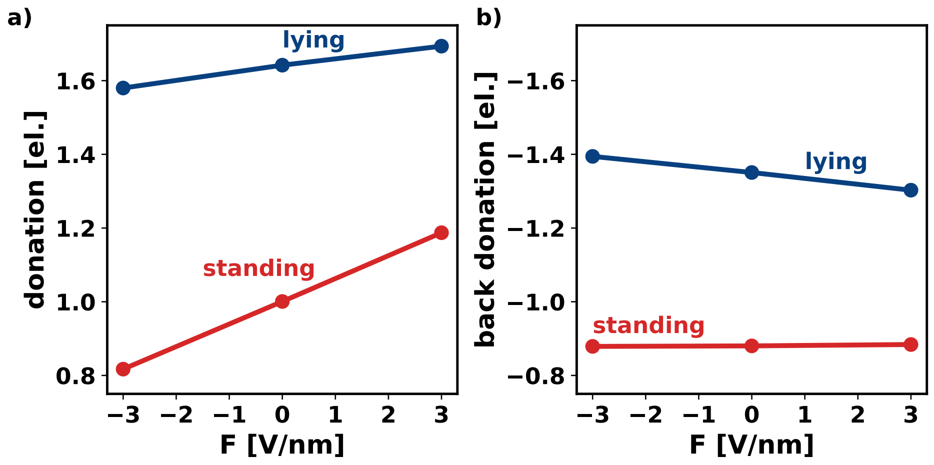

Figure S6: Components of the charge transfer for different electric fields F. a) Forward donation (from substrate to molecule). b) Backward donation (from molecule to substrate)

S4 Charge density difference for $F = -3\,\mathrm{V\,nm^{-1}}$

Figure S7 shows that change of charge density for isolated molecules on the surface due to an electric field of $F = -3\,\mathrm{V\,nm^{-1}}$. The plot is analogous to Figure 3a in the main paper, where the charge difference is shown for $F = +3\,\mathrm{V\,nm^{-1}}$. The redistribution is qualitatively very similar (except for the opposite sign).

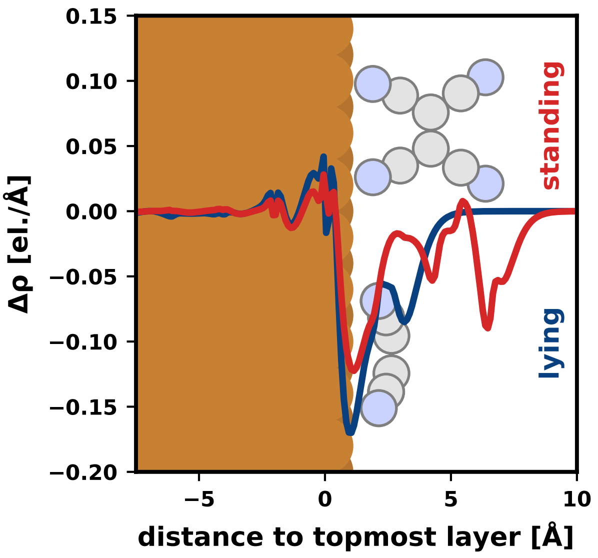

Figure S7: Change in charge density due to an electric field of $F = -3\,\mathrm{V\,nm^{-1}}$, compared to the same system without an electric field applied.

S5 Details on SAMPLE (settings & hyperparameters) and comparison with earlier work

S5.1 Structure search without electric field

We employ the SAMPLE approach[6] to perform interface structure search. SAMPLE is a machine learning based tool that exhaustively generates all commensurate polymorphs and then predicts their energies using Bayesian linear regression and a small number of band-structure calculations. The polymorphs are generated by combination of building blocks in the form of isolated molecule adsorption geometries. As explained in the main paper, these adsorption geometries are obtained by relaxing TCNE molecules from various starting points in a 6×6 supercell, where the molecule-molecule interactions become negligible. A detailed explanation of the SAMPLE process is given in Hörmann et al.[6] and the Supporting Information of Egger et al.[1]

SAMPLE predicts the monolayer adsorption energy $E_{\mathrm{ads}}^{\mathrm{ML}}$ of polymorphs using an energy model that describes the energy as a sum of one-body (molecule-substrate) and two-body (molecule-molecule) terms:

$$E_{\mathrm{ads}}^{\mathrm{ML}} = \sum_i n_i U_i + \sum_j m_j V_j, \tag{S1}$$

where U_i are the one-body terms, V_j are the two-body terms and n_i and m_j describe how often these terms occur in a polymorph. This model is trained using Bayesian linear regression.

We generate more than 13 million polymorphs in a coverage window of 0.9 to 5.1 TCNE nm^{-1} from the standing and lying adsorption geometries shown in Figure S2. We note that "mixed" polymorphs consisting of both standing and lying adsorption geometries were not included because they have been shown to be energetically irrelevant for the polymorphism of TCNE/Cu(11) by Egger et al.[1] Using experimental design theory[6, 7] we draw 100 lying and 199 standing polymorphs and perform band structure calculations with FHI-aims[8] using the PBE functional[9] with MBD-NL dispersion correction.[10] We use 80 test the model predictions. The training is done independently for lying and standing polymorphs, resulting in two sets of SAMPLE hyperparameters shown in Table S1. For more details on the training and the hyperparameters we refer to the original publication.[6] Using the hyperparameters listed in Table S1, we achieve a root mean square error of 14 meV for the lying polymorphs and 22 meV for standing polymorphs on the test set.

Table S1: SAMPLE hyperparameters used to train the SAMPLE energy model for the adsorption energy of lying and standing polymorphs.

Parameter	Lying	Standing
prior one-body interaction uncertainty	0.2 eV	0.2 eV
prior two-body interaction uncertainty	0.1 eV	0.1 eV
real space decay length	0.3 nm	0.3 nm
model uncertainty	0.01 eV	0.01 eV
feature correlation length	1.0	1.0
decay power	−2	−2
feature threshold	0.02	0.02
feature dimension	4	8
minimal intermolecular distance	0.26 nm	0.26 nm
maximal intermolecular distance	1.6 nm	1.6 nm

S5.2 Comparison to Egger et al.

For comparison, we reproduce the results of the structure search performed by Egger et al. in Table S2, with the energies we would predict for these structures. The most stable structures Egger et al. obtained exhibit the same coverage as the ones we present in the current work and are very close in energy to the most stable structures we found. We note in passing that there is only limited meaning to the qualifier "the most stable", as usually several structures with very similar adsorption energies can be found within the prediction uncertainty.[6] The structures found by Egger et al. are the 8th and 3rd best structures in our structures search for lying and standing respectively.

Table S2: The most stable lying and standing structure (regarding energy per area) found by Egger et al. and their properties. We predicted the energies using the SAMPLE model using the settings described above. The rank states the position in an "energy ranking" where all structures generated by SAMPLE are ranked by the energy per area predicted by SAMPLE (using our data and hyperparameters).

Name	Lying	Standing
Depiction	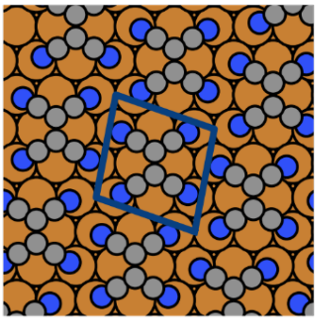	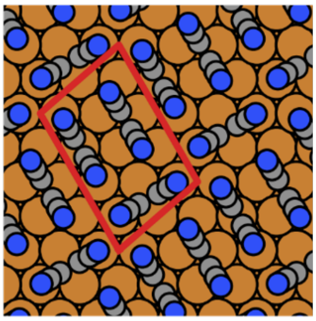
Coverage	2.23 TCNE nm^{-2}	4.46 TCNE nm^{-2}
$E_{\text{ads}}^{\text{SAMPLE}}$ (per molecule)	-1.72 eV TCNE^{-1}	-1.53 eV TCNE^{-1}
$E_{\text{ads}}^{\text{SAMPLE}}$ (per area)	-3.84 eV nm^{-2}	-6.81 eV nm^{-2}
Rank	8th	3rd

Table S3: The number of polymorphs for which we performed band-structure calculates in the respective electric fields F.

Electric field F [V nm^{-1}]	Lying	Standing
-3	50	199
-2	100	249
-1	100	224
$+1$	100	224
$+2$	100	299
$+3$	50	199

S5.3 The SAMPLE approach with electric field

We extend the SAMPLE approach to perform structure search in the electric field. As shown above in Section S2, the adsorption geometries that make up the building blocks SAMPLE uses to generate polymorphs change negligibly for the investigated field strengths. We can therefore use the previously (without any field) generated polymorphs for structure search in the field. We predicted the energy of these polymorphs in electric fields of −3, −2, −1, +1, +2 and $+3\,\mathrm{V\,nm^{-1}}$ by training the SAMPLE model individually for each of the field strengths. To do so we performed band-structure calculations for selected polymorphs with an electric field applied to be used as training and test data. The polymorphs we again selected using experimental design theory. The number of selected polymorphs for which we performed band structure calculations is given in Table S3. 80 % of the polymorphs in the respective field strengths are used for training the corresponding SAMPLE energy models, the remaining 20 % are used to evaluate the performance of the trained models. We used the SAMPLE hyperparameters listed in Table S1 for all field strengths. The root mean squared errors obtained on the test sets for the respective fields are shown in Figure S8.

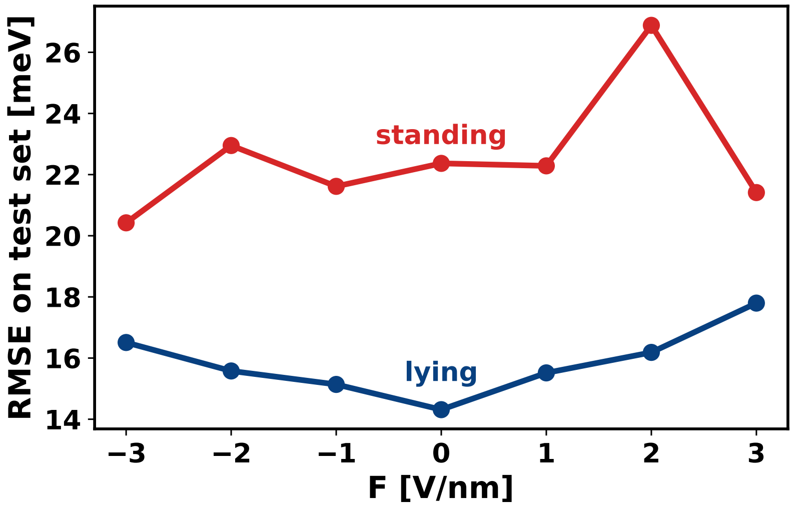

Figure S8: The root mean squared errors (RMSE) for the predictions with SAMPLE on the test set as a function of the field F.

S5.4 Impact of SAMPLE prediction error for the phase boundary

The adsorption energies used to calculate the Gibbs free energy of adsorption for the polymorphs and ultimately the phase boundaries in Figure 6 in the main manuscript are potentially subject to a prediction error (see Figure S8). Therefore, an analysis for the sensitivity of the location of the phase boundaries to changes in the SAMPLE prediction of the adsorption energy was performed. Therein, we added/subtracted the test RMSE (see Figure S8) to the predicted adsorption energies such that the energy difference between lying and standing polymorphs is maximized. The resulting shifted phase boundaries are shown as error bands in Figure S9.

S6 Discussion of the behavior of $E^{\mathrm{ML}}_{\mathrm{formation}}$ as a function of the field

For the monolayer formation energy $E^{\mathrm{ML}}_{\mathrm{formation}}$, the adsorbate dipole $p^{\mathrm{ML}}_{\mathrm{ads}}$ (cf. Figure S13) plays an important role. Figure S10 shows how the formation energy changes with the field.

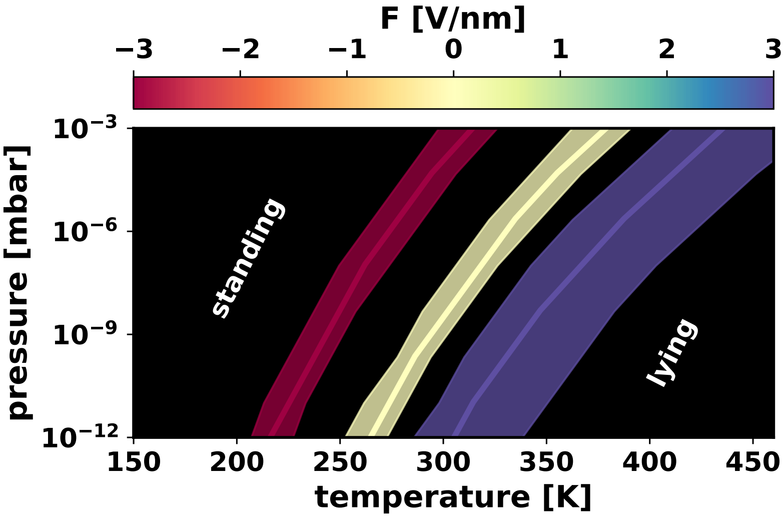

Figure S9: Sensitivity of the phase boundaries to errors in the prediction of the adsorption energy via SAMPLE.

We find that the formation energy is positive (i.e., repulsive) for all investigated electric field strengths. Firstly, this implies that the (deformed) molecules repel each other due to the tight packing. Secondly, the formation energies of lying and standing polymorphs show different behavior regarding the direction of the field. For the standing polymorph, the formation energy of the monolayer becomes more positive for both field directions. For the lying polymorph, however, $E^{\mathrm{ML}}_{\mathrm{formation}}$ is even decreased for $F > 0\,\mathrm{V\,nm^{-1}}$. This behavior is caused by the (repulsive) interaction of adsorbate dipoles. The standing geometries have an a-priori (i.e., for $F = 0\,\mathrm{V\,nm^{-1}}$) very small adsorbate dipole $p^{\mathrm{ML}}_{\mathrm{ads}}$. This is because their geometry deviates only very little from the relaxed TCNE molecule in gas phase. When an electric field is applied standing molecules are polarized. The resulting dipole is nearly the same in magnitude for both field directions ($F < 0\,\mathrm{V\,nm^{-1}}$and $F > 0\,\mathrm{V\,nm^{-1}}$), but differently oriented. This is because the initial adsorbate dipole is immediately compensated by charge rearrangements due to an electric field in the opposing direction. The polarization translates to $p^{\mathrm{ML}}_{\mathrm{ads}}$ increasing with increasing field strengths for the standing polymorph. However, the adsorbate dipoles of the individual adsorbate molecules repel each other, causing increasing formation energies for larger field magnitudes. On the other hand, the lying polymorphs exhibits a significant adsorbate dipole in absence of electric fields. For $F > 0\,\mathrm{V\,nm^{-1}}$ this dipole is reduced by the electric field because the molecule is (de-)polarized. This reduces the repulsive dipole-dipole interaction, causing a reduction in monolayer formation energy. For $F < 0\,\mathrm{V\,nm^{-1}}$ the opposite is the case: The polarization increases the net adsorbate dipole due to polarization along the dipole axis. This increases the repulsive interaction and yields more positive monolayer formation energies. The adsorbate dipoles for the discussed polymorphs are shown together with the interface and bond dipoles in Figure S13.

Even though $E^{\mathrm{ML}}_{\mathrm{formation}}$ is small compared to the other components of $E^{\mathrm{ML}}_{\mathrm{ads}}$ for the fields we investigated, we expect it to become more relevant for even stronger field intensities. As the standing geometries exhibit larger dipoles, and the repulsive dipole-dipole interaction increases $E^{\mathrm{ML}}_{\mathrm{formation}}$ we expect this effect to destabilize standing polymorphs, making them less favorable than lying polymorphs in this regime.

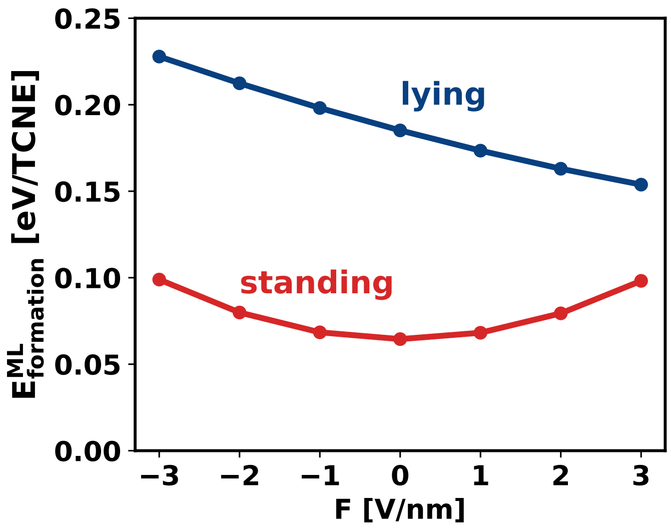

Figure S10: Monolayer formation energy of the most stable (regarding energy per area) lying and standing polymorph we obtained.

S7 Components of $E^{\mathrm{ML}}_{\mathrm{bond}}$ and their evolution with the field

For the following discussion, the definition of an embedding energy is helpful. The embedding energy E_{emb} describes the energy of a charge distribution ρ in the potential Φ resulting from the electric field $\mathbf{F}$:

$$E_{\mathrm{emb}} = -\int \Phi(\mathbf{F})\rho \, \mathrm{d}V \tag{S2}$$

The electric field $\mathbf{F}$ is homogenous in the unit cell and perpendicular to the substrate surface. I.e., $\mathbf{F} = \mathbf{e}_z F$, where $\mathbf{e}_z$ is the unit vector in the direction perpendicular to the surface (pointing away from the surface) and F is the magnitude of the field. As a result, $\Phi(x, y, z) = F \cdot z$, z is the coordinate on the $\mathbf{e}_z$ axis of the system, and x, y are the coordinates of the axes in the plane of the substrate surface. Inserting this potential into Equation S2 we obtain

$$E_{\mathrm{emb}} = -F \int \rho(z) z \, \mathrm{d}z \tag{S3}$$

To investigate how much of the monolayer bonding energy $E^{\mathrm{ML}}_{\mathrm{bond}}$ stems from the embedding energy we separate the bonding energy into a field dependent and a field independent part

$$E^{\mathrm{ML}}_{\mathrm{bond}}(F) = E^{\mathrm{ML}}_{\mathrm{bond}}(F = 0) + \Delta E^{\mathrm{ML}}_{\mathrm{bond}}(F). \tag{S4}$$

We further decompose $\Delta E^{\mathrm{ML}}_{\mathrm{bond}}(F)$ into a part that corresponds to the change in the embedding energy (E_{emb}, due to adsorption of monolayer on the substrate) and a rest that corresponds to charge redistribution in order to minimize the embedding energy (E_{pol}):

$$\Delta E^{\mathrm{ML}}_{\mathrm{bond}}(F) = E_{\mathrm{emb}}(F) + E_{\mathrm{pol}}(F). \tag{S5}$$

For the structures discussed in Section S2.2, the change of the monolayer bonding energy with the field $\Delta E^{\mathrm{ML}}_{\mathrm{bond}}$ and its components E_{emb} and E_{pol} are shown in Figure S11a-S11c. We find that the majority of $\Delta E^{\mathrm{ML}}_{\mathrm{bond}}$ stems from E_{emb}, which is almost ten times as large as E_{pol}. In accordance with the discussion in the main paper, we also find that the embedding energy varies more strongly for the standing geometry. E_{pol}, on the other hand, changes more for the lying geometry.

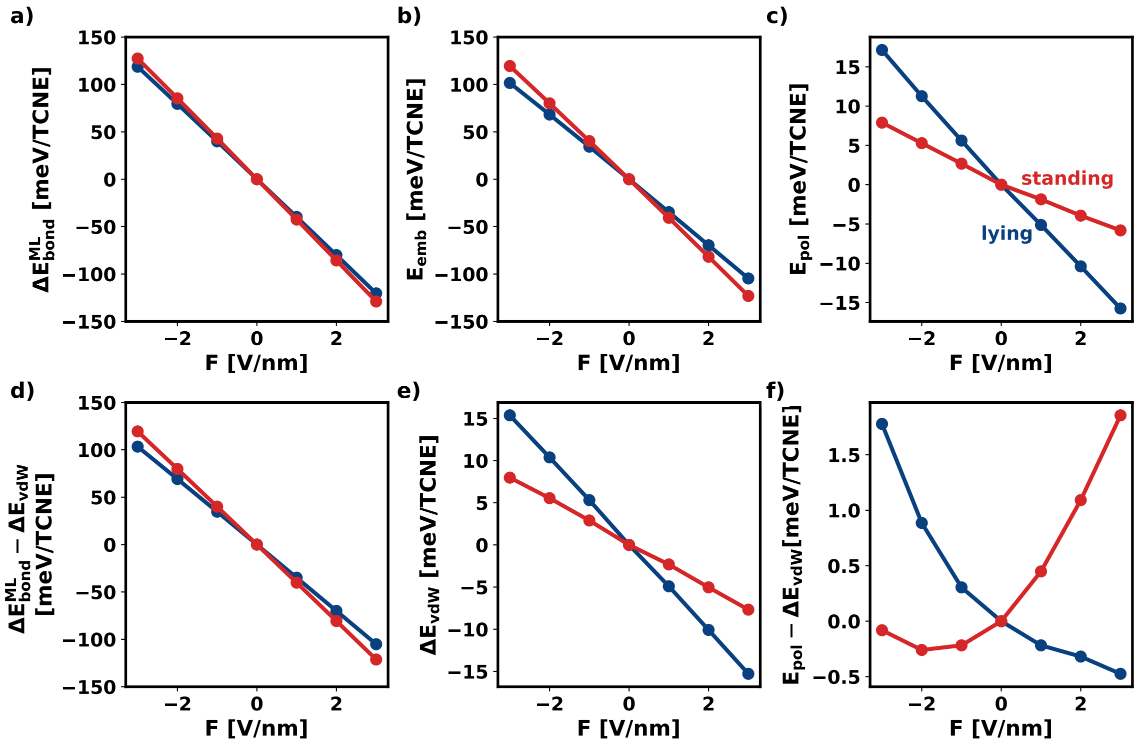

Figure S11: Components of the monolayer bonding energy as a function of the electric field F. a) monolayer bonding energy, b) embedding energy c) energy due to redistribution of charges d) difference monolayer bonding energy and its van der Waals component e) van der Waals component of the monolayer bonding energy f) difference energy due to redistribution of charges and the van der Waals component of the monolayer bonding energy.

Finally, we analyze the influence of van der Waals interactions on the change of the bonding energy with the field. We assume that the redistribution energy E_{pol} has a component ΔE_{vdW} that accounts for the all the change in the van der Waals component of E_{bond} due to the adsorption process. Figure 11e shows ΔE_{vdW} as a function of the field. We find that it makes up most of E_{pol}, with the absolute difference (shown in Figure S11f) being less than 2 meV for all investigates field strengths. Furthermore, we observe that ΔE_{vdW} is only a small component of $\Delta E^{\mathrm{ML}}_{\mathrm{bond}}$. Figure S11d shows the difference of the two components.

S8 Adsorption energy and its components per area

In the main paper, we discuss the monolayer adsorption energy and its components in terms of energy per molecule. For the interested reader, we also provide them in energy per area in Figure S12.

S9 The interface dipole and its components

To get a give a more direct understanding of the embedding energies in the system, we calculated the interface dipole $p^{\mathrm{ML}}_{\mathrm{int}}$ and its components for the polymorphs discussed in Section 2.3 in the main paper. We do this by numeric integration of the charge density computed by FHI-aims at various fields F between $-3\,\mathrm{V\,nm^{-1}}$ and $+3\,\mathrm{V\,nm^{-1}}$. Accordingly, we integrated

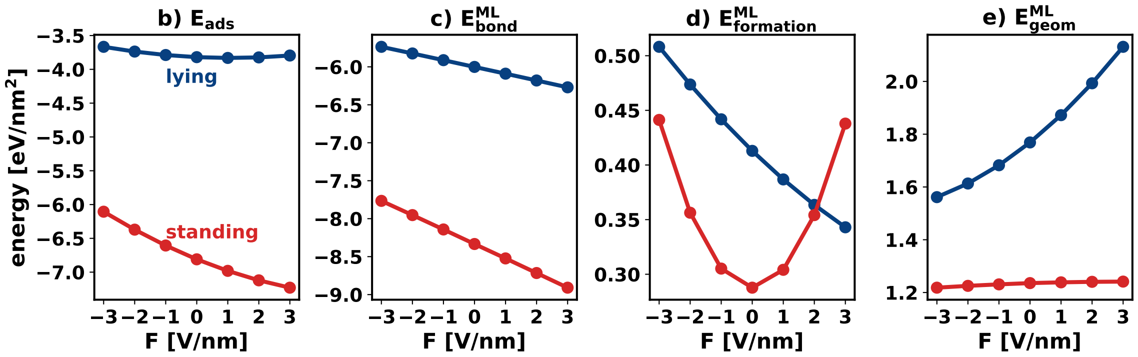

Figure S12: Monolayer adsorption energy and its components as a function of the electric field F. All values are given in energy per area. a) monolayer adsorption energy b) monolayer bonding energy c) monolayer formation energy d) monolayer deformation energy.

the charge density of the freestanding adlayer in vacuum to obtain the adsorbate dipole p^{ML}_{ads} Simple subtraction yields the $p^{ML}_{bond} = p^{ML}_{int} - p^{ML}_{ads}$. The result is shown in Figure S13.

The slope of p^{ML}_{ads} reflects the average polarizability of the adsorbate molecules in the lying and standing polymorphs. As was to be expected from gas phase results, the standing molecules are polarized more easily. We find that without an external electric field p^{ML}_{ads} is very small for standing. This is because the adsorbate geometry is comparably close the geometry of the molecule in vacuum. However, when a field is applied the standing adsorbate molecules become polarized and subsequently yield an adsorbate dipole opposed to the direction of the field. Lying, on the other hand, exhibits a significant adsorbate dipole due to its geometry, which is increased or decreased by the field due to additional polarization of the molecule.

As opposed to this, the p^{ML}_{bond} changes very little with the field for both categories. We find that the bond dipole is larger for the standing polymorph. This agrees with our findings from Section 2.3 where we investigated how the charge density of the interface systems is affected by the field. There, we found that additional charge transferred into the adsorbates is located further away from the surface (which also implies a larger bond dipole).

Combining adsorbate and bond dipole, we find p^{ML}_{int} changes almost exactly with the slope of the adsorbate. As p^{ML}_{bond} and p^{ML}_{ads} mostly cancel each other due to their opposite directions, we find that the resulting interface dipole for standing is significantly larger than for the lying polymorph. In particular, the interface dipole of lying even changes in direction as it is overcompensated by the adsorbate.

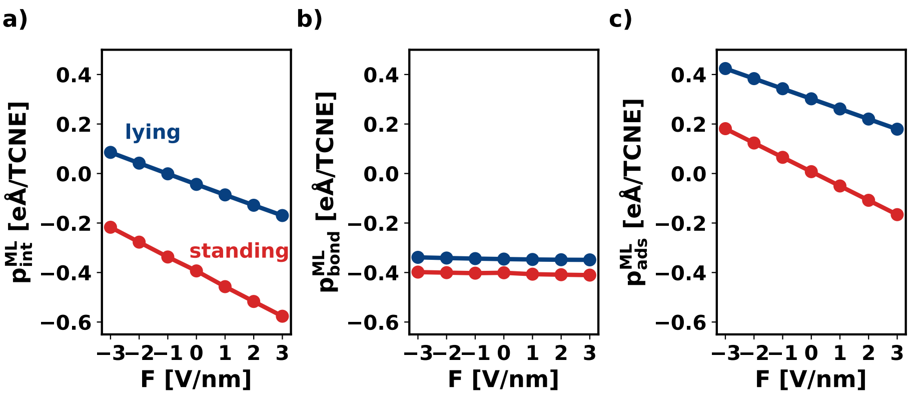

Figure S13: Interface dipole and its components as a function of the electric field. a) Interface dipole, b) bond dipole c) adsorbate dipole.

S10 How does the electric field change the vibrations of the interface?

In the following discussion we argue that the field affects the vibrational energies of TCNE/Cu(11) only very little. We focus on the zero-point energy of the vibrations. As the temperature dependence of the vibrational energies is usually similar between different polymorphs,[1] we expect temperature effects to remain negligible in the electric field as well.

Because vibrational calculations for all relevant interface systems are intractable, we approximate the vibrations of adsorbates on the surface using molecules in gas phase.

S10.1 Different orientations in the electric field

Given the anisotropic polarizability of TCNE, one might expect its vibrational spectrum to be affected differently for different orientations of its π-plane to an external electric field. To investigate this, we relaxed a TCNE molecule in gas phase with i) its z-axis, ii) its y-axis, or iii) its z-axis perpendicular to the field until the forces fell below $1 \times 10^{-3}\,\mathrm{eV\,Å^{-1}}$. For all orientations the geometry changes negligibly. Using the relaxed geometries, we performed a vibrational analysis. We approximate the Hessian via finite differences using displacements of 0.0025 Å. Figure S14 shows the zero-point energy of TCNE in different orientations to the field as a function of field strength F. The sign of F represents the direction of the field (cf. main paper).

We find that the zero-point energy changes only very little with the electric field for either orientation. As was to be expected, the TCNE molecule with its π-plane aligned with the field (y in Figure S14) is more strongly affected. However, even in this case the zero-point energy changes by less than 2.5 meV. We conclude that the change in the vibrational spectrum of TCNE due to polarization caused by an electric field is negligible.

S10.2 Different charge states

As discussed in the main paper, one of the effects of the electric field is the change in charge transfer from the substrate into the LUMO of the adsorbates. As shown in the MODOS calculation above, the back donation is mostly unaffected by the field. As was discussed in previous work,[1, 11] the charge transfer has a strong impact on the vibrational spectrum of the interface system. This is, e.g., due to the change in C=C bond strength in the TCNE backbone. To approximate these changes in the vibrational degrees of freedom with changes

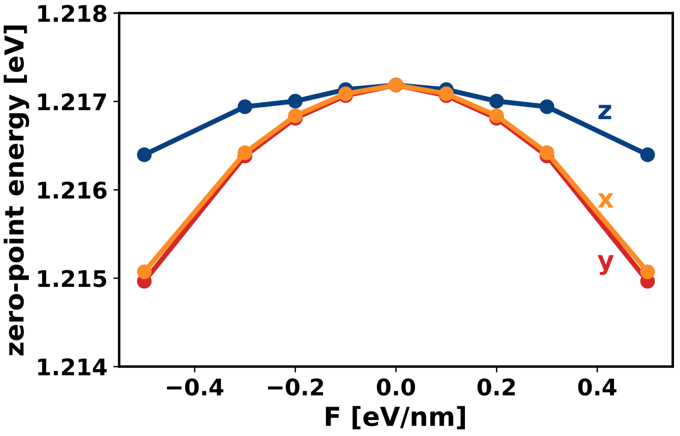

Figure S14: Zero-point energy of a TCNE molecule in vacuum as a function of the field F for different orientations: x, y, and z correspond to the respective axis of the molecule being parallel to field.

in charge transfer due to the field, we investigate the change in vibrations in an isolated TCNE molecule when charged.

We relaxed the molecule for every charge state until the forces fell below $1 \times 10^{-3}\,\mathrm{eV\,Å}^{-1}$ and perform vibrational analysis with a Hessian we calculated using finite differences. We used displacements of 0.0025 Å. We find that the zero-point energy of the molecules changes linearly with the amount of additional charge as is shown in Figure S15.

S11 How does the electric field change the vibrations of the interface?

In the following we discuss how the electric field affects various degrees of freedom of TCNE in gas phase that are relevant for the calculation of the Gibbs free energy of the gas phase.

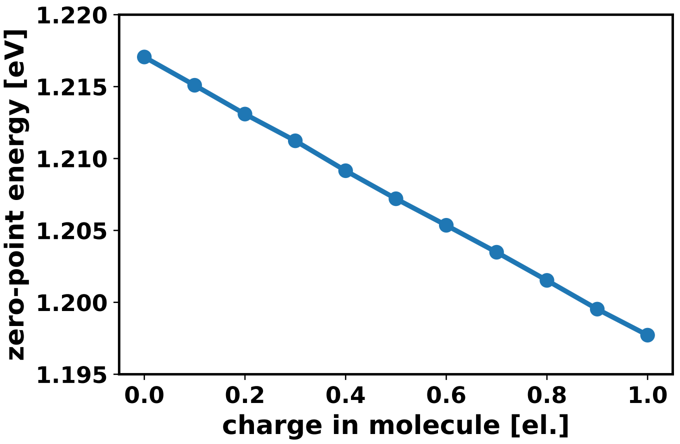

Figure S15: Zero-point energy of a TCNE molecule in vacuum as a function of different levels of charging.

S11.1 Electronic degrees of freedom: orientation of the molecule

Due to the anisotropic polarizability, TCNE exhibits an orientation dependent electronic energy in the electric field. We calculated the energy of a TCNE molecule in vacuum ($E^{\mathrm{vac}}_{\mathrm{mol}}(\Omega)$) in 441 different orientations Ω for electric fields F between $-3\,\mathrm{V\,nm^{-1}}$ and $+3\,\mathrm{V\,nm^{-1}}$ using FHI-aims. The sign corresponds to the direction of the field. The orientations are obtained by applying a 21 azimuthal and 21 polar rotations. Figure S16 shows the difference $\Delta E^{\mathrm{vac}}_{\mathrm{mol}}(\Omega)$ between the most favorable orientation Ω_{best} and the least favorable orientation Ω_{worst}

$$\Delta E^{\mathrm{vac}}_{\mathrm{mol}}(F) = E^{\mathrm{vac}}_{\mathrm{mol}}(F, \Omega_{\mathrm{best}}) - E^{\mathrm{vac}}_{\mathrm{mol}}(F, \Omega_{\mathrm{worst}}) \tag{S6}$$

for different fields F. As was to be expected the difference between best and worst orientation increases with the magnitude of the field (irrespective of field direction). However, for all fields investigated, $\Delta E^{\mathrm{vac}}_{\mathrm{mol}}$ remains relatively small, amounting to approximately 35 meV for $F = \pm 3\,\mathrm{V\,nm^{-1}}$.

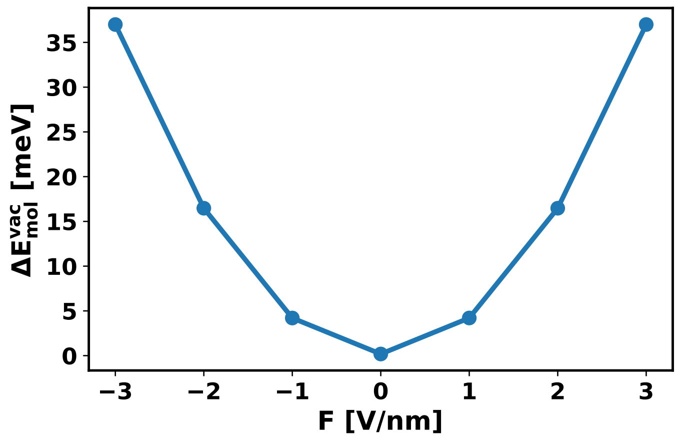

Figure S16: Orientation dependence of TCNE in the electric field F. Difference of the energy of the molecule in the most preferential and the most unfavorable orientation.

In fact, when thermally occupying all the different orientations, we find that the change in expected molecule energy due to the other orientations changes by at most 15 meV for 400 K for the strongest fields we employed as can be seen in Figure S17.

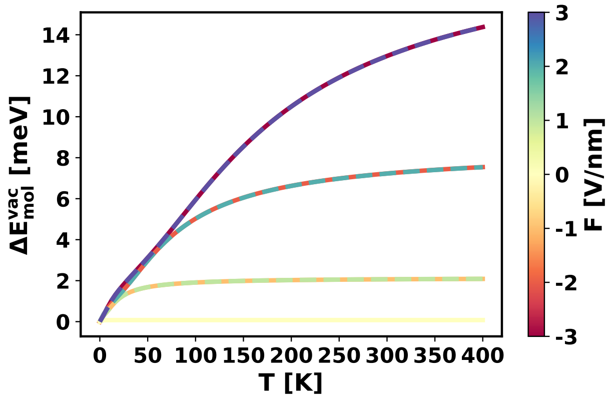

Figure S17: Change in the energy of TCNE in vacuum for different electric field as a function of temperature.

S11.2 Rotational degrees of freedom

Given the rotation dependence of the electronic energy of TCNE in an electric field, the rotation of the molecule becomes hindered. This is equivalent to the molecule – in addition to its rotation – oscillating in a potential, the depth of which is given by $\Delta E_{\mathrm{mol}}^{\mathrm{vac}}(F)$. The potential is depicted in Figure S18, where we represented Ω using two angles of rotation $\Omega = (\phi, \theta)$. Upon closer inspection, we find that the change in potential is very small in ϕ-direction. Therefore, we neglect the field dependence in ϕ direction, which reduced the hindrance of the rotation to just one rotational dimension: θ.

To investigate the significance of the hindrance, we use a finite differences approach to solve the one-dimensional Schrödinger equation for the potential given by $\Delta E_{\mathrm{mol}}^{\mathrm{vac}}(\theta)|_{F=3\,\mathrm{V\,nm^{-1}}}$. We find that the zero-point energy of this hindered rotation is below 0.5 meV. Therefore, we can safely neglect the impact of the electric field for the rotation of the TCNE molecules in gas phase.

S11.3 Vibrational degrees of freedom

We already discussed how the electric field affects the vibrations of TCNE in vacuum when discussing the vibrational energies of the interface system in Section S10.1. We find that the zero-point energy changes very little with the field (for the field strengths we investigated).

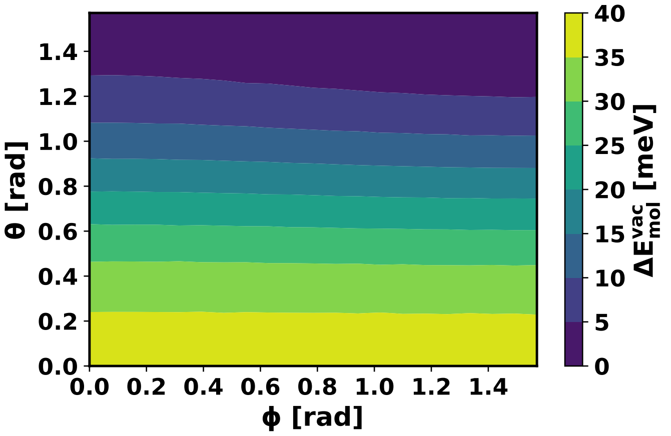

Figure S18: Energy of the molecule as a function of its orientation for an external electric field of $F = 3\,\mathrm{V\,nm^{-1}}$.

S12 References

(1) Egger, A. T.; Hörmann, L.; Jeindl, A.; Scherbela, M.; Obersteiner, V.; Todorović, M.; Rinke, P.; Hofmann, O. T. *Advanced Science* **2020**, *7*, 2000992.

(2) Hörmann, L.; Jeindl, A.; Hofmann, O. T. *The Journal of Chemical Physics* **2020**, *153*, 104701.

(3) Hörmann, L.; Jeindl, A.; Hofmann, O. T. *The Journal of Chemical Physics* **2022**, *156*, 206101.

(4) Werkovits, A.; Jeindl, A.; Hörmann, L.; Cartus, J. J.; Hofmann, O. T. *ACS Physical Chemistry Au* **2022**, *2*, 38–46.

(5) Rangger, G. M.; Romaner, L.; Heimel, G.; Zojer, E. *Surface and Interface Analysis* **2008**, *40*, 371–378.

(6) Hörmann, L.; Jeindl, A.; Egger, A. T.; Scherbela, M.; Hofmann, O. T. *Computer Physics Communications* **2019**, *244*, 143–155.

(7) Wald, A. *The Annals of Mathematical Statistics* **1943**, *14*, 134–140.

(8) Blum, V.; Gehrke, R.; Hanke, F.; Havu, P.; Havu, V.; Ren, X.; Reuter, K.; Scheffler, M. *Computer Physics Communications* **2009**, *180*, 2175–2196.

(9) Perdew, J. P.; Burke, K.; Ernzerhof, M. *Physical Review Letters* **1996**, *77*, 3865–3868.

(10) Hermann, J.; Tkatchenko, A. *Physical Review Letters* **2020**, *124*, 146401.

(11) Erley, W.; Ibach, H. *The Journal of Physical Chemistry* **1987**, *91*, 2947–2950.
